# Converting melanoma-associated fibroblasts into a tumor-suppressive phenotype by increasing intracellular Notch1 pathway activity

**DOI:** 10.1371/journal.pone.0248260

**Published:** 2021-03-11

**Authors:** Hongwei Shao, Mecker Moller, Long Cai, Rochelle Prokupets, Cuixia Yang, Connor Costa, Kerstin Yu, Nga Le, Zhao-Jun Liu

**Affiliations:** Department of Surgery, University of Miami School of Medicine, Miami, Florida, United States of America; Duke University School of Medicine, UNITED STATES

## Abstract

Cancer-associated fibroblasts (CAFs) play a crucial role in cancer progression, drug resistance and tumor recurrence. We have recently shown that the Notch pathway determines the tumor-regulatory role of experimentally created ‘CAFs’. Here, we examined the status of Notch signaling in human melanoma-associated fibroblasts (MAFs) *versus* their normal counterparts and tested whether manipulation of the Notch pathway activity in MAFs alters their tumor-regulatory function. Using tissue microarrays, we found that MAFs exhibit decreased Notch pathway activity compared with normal fibroblasts in adjacent and non-adjacent skin. Consistently, MAFs isolated from human metastatic melanoma exhibited lower Notch activity than did normal human fibroblasts, demonstrating that Notch pathway activity is low in MAFs. We then investigated the effect of increasing Notch pathway activity in MAF on melanoma growth in co-cultures and in a mouse co-graft model. We found that activation of the Notch pathway in MAFs significantly restricted melanoma cell growth *in vitro* and suppressed melanoma skin growth and tumor angiogenesis *in vivo*. Our study demonstrates that the Notch signaling is inhibited in MAFs. Increase of Notch pathway activity can confer tumor-suppressive function on MAFs. Thus, targeting melanoma by activating Notch signaling in MAF may represent a novel therapeutic approach.

## Introduction

Tumor stromal fibroblasts, also known as cancer-associated fibroblasts (CAFs), are the major cellular components of the reactive tumor stroma. CAFs and tumor cells co-evolve as critical components in the regulation of tumor progression, by eliciting a variety of ECM remodeling enzymes and exosomes, structural components of the extracellular matrix (ECM), and soluble factors [1, 2]. CAFs also influence cancer organ-specific metastasis [3]. Tumor cell survival and regrowth in the parenchyma of foreign tissues is supported by transport of CAF assisted tumor cells disseminating from primary lesions, to the metastatic niche [4]. Additionally, CAFs play a part in drug resistance and tumor recurrence [5, 6]. It remains unknown how CAFs precisely accomplish tumor-regulation, despite the extensive evidence supporting CAFs involvement. We have recently demonstrated that the tumor regulating function of CAFs are governed by the Notch1 signaling pathway.

The Notch pathway determines a variety of cellular activities in different types of cells [7, 8]. We and others previously observed a correlation between the status of Notch signaling and activity of fibroblasts. Constitutive activation of the *Notch1* pathway slowed cell motility and growth of human fibroblasts, while Loss of Notch1 in mouse embryonic fibroblasts (MEFs) conferred faster cell motility rate and growth [9]. Cell‐cycle arrest and apoptosis in MEFs are consistently observed after Notch activation [10]. These studies indicate that fibroblast cell growth and cellular activity is negatively regulated by the activation of the Notch cell signaling pathway. Quiescent and proliferating human dermal fibroblasts exhibit striking differential patterns during analysis of Notch pathway gene expression profiling. Quiescent fibroblasts manifest increased levels Notch pathway characteristic gene components, whereas proliferating fibroblasts express low or undetectable levels [9]. These findings suggest that quiescent fibroblasts experience Notch signal activation or high levels of activity, whereas proliferating fibroblasts experience inactivation of low levels of Notch activity. In addition, inhibited melanoma growth and angiogenesis in the xenograft model [11] (co-grafted normal skin fibroblasts, pre-engineered to carry high Notch1 activity) reveal that the tumor-promoting effect of stromal fibroblasts is antagonized by Notch activation. Using novel mouse models established in our lab, in which genetic Notch 1 signal activation or inactivation specifically occurring in natural host fibroblast cells, we consistently demonstrated that melanoma invasion was promoted by CAFs carrying null Notch1 while those with significantly inhibited melanoma growth and invasion expressed elevated Notch1 activity [12]. These results indicated a crucial role for Notch signaling in governing tumor regulatory function of CAFs. However, the fibroblasts investigated in these earlier studies are experimentally created artificial ‘CAFs’, and their intracellular Notch signaling pathway activities are experimentally modified. In this study, we assessed the intracellular Notch signaling activity in natural human melanoma-associated fibroblasts (MAFs) *versus* their normal counterparts and tested whether manipulation of the Notch pathway activity in MAFs alters their tumor regulatory function.

## Materials and methods

### Cells, cell proliferation and viability assays

Primary human dermal fibroblasts (FF2441) were isolated from neonatal foreskin and obtained from the Wistar Institute [13]. Normal human dermal fibroblasts from adults (NHDF-Ad) were purchased from Lonza (CC-2511). Human MAFs were isolated from three human skin melanoma specimens, which were collected from three adult patients who were above 18 years old, according to the principles expressed in the Declaration of Helsinki and using the protocol approved by the committee of institutional review board (IRB) of the University of Miami (IRB # 20100200). Written informed consent was obtained from the parents before surgery. Patients’ identities are anonymous. The method to isolate human cells were carried out in accordance with the guidelines and regulations of using human tissues by the IRB of the University of Miami. NHDF-Ad was cultured in FGM™-2 BulletKit™ (Lonza, NJ). All other fibroblasts were cultured in Dulbecco’s Modified Eagle’s Medium (DMEM) supplemented with 20 mM L-glutamine, 8 mM HEPES and 10% FBS (Hyclone, Logan, UT). Human melanoma cell lines derived from vertical growth phase (WM278 [14]) and metastatic phase (1205Lu [15] and C8161 [16]) were obtained from the Wistar Institute and cultured in 2% FBS-W489 medium as described [15]. All cell culture media were purchased from Invitrogen (Carlsbad, CA) unless indicated. All cells underwent mycoplasma testing in our lab and were mycoplasma free.

Cell growth and effect of CM on cell growth were tested using WST cell proliferation kits (BioVision, Mountain Views, CA) as described [11]. Cell viability of various MAFs was tested by co-staining cells with TUNEL staining (TUNEL Assay Kit—In situ BrdU-Red DNA Fragmentation, ab66110, Abcam) and anti-active Caspase3 staining (anti-Caspase-3, ab2302; Alexa Fluor 405-conjugated 2nd antibody, ab175649, Abcam) as previously described [11]. For cell growth and viability assays, cells were tested in 3 replicates and assays were repeated three times.

### Cell co-culture of MAF−melanoma

N1^IC^-GFP/MAF or GFP/MAF were mixed with melanoma cells (1205Lu, pre-transduced with DsRed/Lentivirus and sorted by FACS) at a 2:1 ratio and a total of 1.2 x 10^5^ cells/well were plated into 6-well plates. Cells were cultured in serum-free W489 and DMEM mixture (1:1). At days 1–4, portions (individual wells) of solo- and co-cultures were detached for flow cytometry analysis (FACSAria II, BD Biosciences, CA) to determine the numbers of melanoma cells (DsRed^+^) and MAFs (GFP^+^).

### Lentivirus and transduction

Methods for generation of GFP/lentiviral, N1^IC^–GFP/lentiviral, DsRed/lentiviral and Luc2/lentiviral vectors were described previously [11]. Transduced cells were cultured with a regular medium for 3 days, sorted by FACS and tested in subsequent analyses.

### Tissue microarray and immunofluorescence and immunohistochemistry

Each ME482 human melanoma tissue microarray slide (#063, #064, #065, US Biomax, Rockville, MD) contains 48 cases/48 cores of malignant melanoma with matched normal tissue control (*http*:*//www*.*biomax*.*us/tissue-arrays/Melanoma/ME482*). According to US Biomax, human melanoma tissues and normal tissues used to prepare for microarray slides were derived from adult (>18 years old) patients or donor from whom written informed consents were obtained. To do IF, the sections were deparaffinized, rehydrated prior to antigen retrieval. Slides were incubated with Alexa Fluor® 594-conjugated anti-Hes1 (ab71559, Abcam) and FITC-conjugated anti-FSP-1 (fibroblast specific protein-1) (GTX89197, GeneTex) after being blocked with Protein Block (Dako, Carpinteria, CA). Nuclei were stained with DAPI (Sigma-Aldrich, St. Louis, MO). Co-grafted melanoma tissue sections were stained by anti-Ki67 and anti-GFP (ab15580 and ab6658, Abcam) and followed by corresponding secondary antibodies. IHC using anti-CD31 (ab56299, Abcam) and *ApopTag® Plus Peroxidase In Situ Apoptosis Kit* (Millipore Corporation, Billerica, MA) were performed as described [11]. To detect apoptotic tumor cells, co-grafted melanoma tissue sections were co-stained by TUNEL Assay Kit—In situ BrdU-Red DNA Fragmentation, ab66110, Abcam) and anti-Luciferase as tumor cells were pre-transduced with Luc/Lentivirus (ab181640, Abcam and Alexa fluor488-conjugated 2^nd^ antibody, A11055, Invitrogen).

### PCR Array and immunoblot

The Human Notch Pathway *RT*^*2*^*-Profiler™ PCR Array* (#PAHS-059, Invitrogene) was conducted as described [9]. All samples were normalized to the levels of *β-actin*, and results are expressed as relative fluorescence intensity. 4%-20% gradient SDS-PAGE gel was used for Hes-1 in Fig 2C-related experiment, while 10% SDS-PAGE gels were used for other Western blot experiments. Immunoblot was performed as described [17]. Membranes were probed with Abs to Notch1 (ab52627, Abcam for Fig 2C; ab27526, Abcam for Fig 2D; and N4788, Sigma-Aldrich for Fig 3A, respectively), Notch4 (TA321658, Origene) that detected full length Notch4 expression in MAFs, activated Notch1 (for Fig 3A, N4788, Sigma-Aldrich), Hes-1 (ab71559, Abcam for Fig 2D and Fig 3A; ab55265, Abcam for Fig 2C, respectively), Hey-1 (GTX42614, GeneTex), WISP-1 (SAB1400324, Sigma-Aldrich) or β-actin (A1978, Sigma-Aldrich) accordingly. Autophotographs of blots were scanned by densitometer (Molecular Dynamics) to quantify the bands.

### MAF−melanoma co-graft mouse model

All animal experiments were approved and performed in the shelters in accordance with animal welfare rules and relevant guidelines and regulations by the University of Miami Institutional Animal Care and Use Committee (IACUC) under protocol # 18–083. A mixture of 2.4 x 10^6^ MAFs and melanoma cells was injected intradermally on the dorsal site of the SCID mice (Charles River, Wilmington, MA) at age 8–10 weeks (male and female are 50%: 50%). Tumor size was recorded by weekly measurement of tumor length and width using a caliper and tumor volume is calculated using the formula: width × width × length / 2. Mice were anesthetized for all surgical procedures by ketamine/xylazine mixture (100/10 mg/kg, IP), and imaging procedures by inhaling 3% isoflurane gas, and sacrificed in CO2 chamber.

### Blood vessel perfusion and confocal microscopy

Blood vessels were labeled by live perfusion using a DiI (D-282, Invitrogen) solution via direct intra-cardiac injection as previously reported [17, 18]. Tumor angiogenesis was visualized by scanning the resected tumors to a depth of 200 μm using laser scanning confocal microscopy (Vibratome, T1000S, Leica Microsystems). Vessel density was quantified assessing total number of Dil-labeled (red) vessels normalized to the entire scanned tumor area using ImageJ software (NIH, Bethesda, MD).

### Statistical analyses

Two-tail student’s *t*-test was used for paired samples and ANOVA followed by post-hoc test for multiple samples. Data are expressed as mean ± standard deviation (SD). Values are considered statistically significant when *p*<0.05.

## Results

### Differential status of Notch signaling in MAFs and normal skin fibroblasts

**Fig 1:**
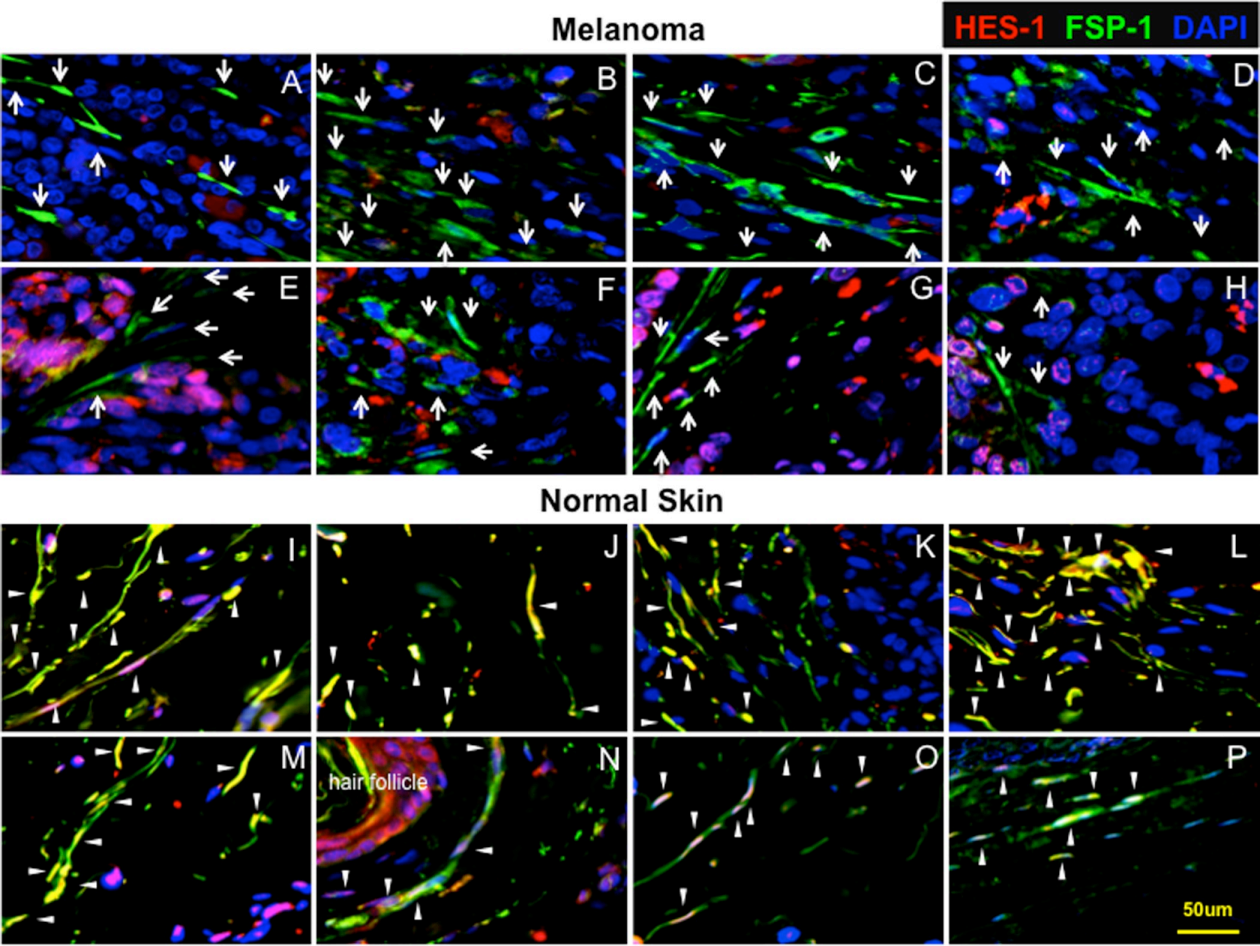
Low Notch activity in MAFs. Sixteen representative IF images of tissue microarrays show that MAFs express lower levels of Hes-1 compared to normal skin fibroblasts. White arrows point to MAFs (*top*), while white arrowheads indicate Hes-1 expression in fibroblasts in normal skin tissues (*bottom*). The strong Hes-1 expression detected in melanoma tissues (*top*) is from malignant melanoma cells in which the Notch pathway is activated, not from MAFs, which are stained as green cells. In normal skin (*bottom*), nuclear Hes-1 is pink as overlapping of blue (DAPI for nuclei) and red (Hes-1), and cytoplasmic is red, because of strong expression of Hes-1 (red). *Top*: A, B, D, E, F, G are melanomas at stage II; C at stage I, and H at stage III. The Notch pathway is activated in melanomas D−H, and is inactivated in melanomas A−C (a few cells stained red in A−C are infiltrated immune cells based on their morphology). *Bottom*: I−M are adjacent skin tissues, while N−P are non-adjacent skin tissues. A single scale bar in a panel of pictures is representative for all pictures.

To study the status of Notch signaling in natural MAFs and their normal counterparts, a tissue microarray approach with immunofluorescence (IF) was undertaken to examine levels of Hes-1, a canonical Notch target, in MAFs of human melanoma at different stages (I-III) and fibroblasts located at adjacent and non-adjacent normal skin tissues. Tissue microarray slides composed of 24 cores derived from melanomas, 9 cores from adjacent and 15 cores from non-adjacent normal skin tissues, were co-stained with anti-Hes-1 and anti-FSP-1 (fibroblast specific protein-1). Compared with adjacent and non-adjacent skin fibroblasts, which exhibited higher levels of Hes-1, expression of Hes-1 was barely detectable in MAFs. The strong Hes-1 expression detected in melanoma tissues (Fig 1, *top*) is from malignant melanoma cells in which the Notch pathway is activated as we reported previously [15], not from MAFs, which are stained as green cells. In normal skin (Fig 1, *bottom*), nuclear Hes-1 is pink as overlapping of blue (DAPI for nuclei) and red (Hes-1), and cytoplasmic is red, because of strong expression of Hes-1 (red). The ratio of Hes-1: FSP-1 in melanomas, adjacent and non-adjacent skin tissues, is 0.077±0.088, 0.537±0.193 and 0.401±0.100, respectively (Table 1). Since the Notch pathway is activated in approximate 50%-60% of melanomas according to our previous study [15], Hes-1 stained positive in some melanoma cells can be used as internal control for MAFs which are FSP-1^+^. Sixteen IF images (8 from melanomas and 8 from adjacent and non-adjacent skin tissues) with the combination of three colors (Hes-1, FSP-1 and DAPI) are shown in Fig 1. See *S1–S3 Figs* for images with individual color. Levels of Hes-1 were undetectable in 18 out of 24 melanomas and marginally measurable in only 6 melanomas (6/24, 25%). There appeared to be no correlation between Hes-1 levels and stages of melanoma malignancy. In contrast, expression of Hes-1 was higher in all adjacent (9/9, 100%) and non-adjacent normal skin fibroblasts (15/15, 100%) (Table 1). These results indicated that low Notch activity is common in MAFs, suggesting that the intracellular Notch pathway is inhibited in MAFs.

**Table 1:** Summary of results of the tissue microarrays. Expression of Hes-1 in fibroblasts at melanoma and normal skin tissues.

	Hes-1^-^/FSP-1^+^ cores/total cores (%)	Hes-1^+^/FSP-1^+^ cores/total cores (%)	Ratio of Hes-1/FSP-1
**Melanoma**	**18/24 (75%)**	**6/24 (25%)**	0.077 ± 0.088**
**Normal skin adjacent to tumor**	**0/9 (0%)**	**9/9 (100%)**	**0.537 ± 0.193**
**Normal skin non-adjacent to tumor**	**0/15 (0%)**	**15/15 (100%)**	**0.401 ± 0.100**

_**_P<0.001 (melanoma vs. adjacent and non-adjacent normal skins, ANOVA)

### The Notch signaling pathway is inhibited in MAFs

**Fig 2:**
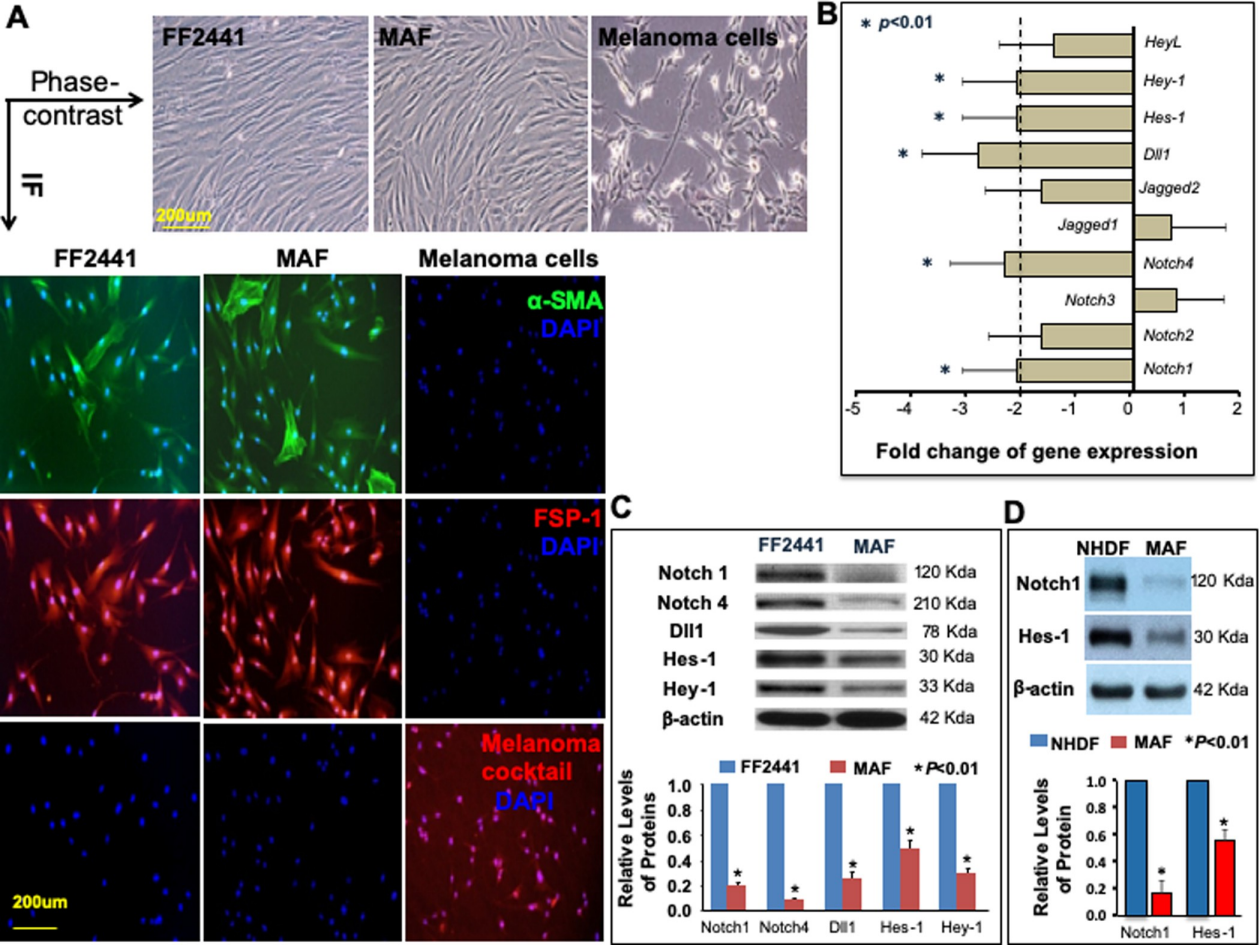
Decreased Notch pathway activity in isolated MAFs. **A.** Characterization of isolated MAFs by morphology (s) and IF (color images), and comparison with normal human skin fibroblasts and melanoma cells. A single scale bar in a panel of pictures is representative for all pictures. **B.** Quantification of gene expression of the Notch pathway components in MAF vs. FF2441 by *PCR Array*. The *Notch1*, *Notch4*, *Dll1*, *Hes-1* and *Hey-1* genes were significantly downregulated (<2-fold) in MAFs (* *P*<0.01). The results are mean ± SD of three independent experiments. **C.** The decreased protein expression of Notch1, Notch4, Dll1, Hes-1 and Hey-1 in MAFs vs. FF2441 was confirmed by immunoblot. Blots were cropped from different gels. β-actin was used as a loading control. **D.** The decreased protein expression of Notch1 and Hes-1 in MAFs vs. NHDF was confirmed by immunoblot. Blots were cropped from different gels. β-actin was used as a loading control. For **C** and **D**, Uncropped images of immunoblot are available in *S1 File*. Relative levels of proteins from three independent Western blots are shown [proteins expressed in normal skin fibroblasts [FF2441 and NHDF, respectively) are set as “1”].

To further evaluate status of Notch signaling in MAFs, we isolated MAFs from human skin melanoma, analyzed the Notch pathway gene expression profiling and compared it with those of normal human skin fibroblasts (FF2441 [13]). Isolated MAF exhibited typical elongated, spindle-shaped fibroblast morphology (Fig 2A, Phase-contrast) and were characterized as α-SMA (α-smooth muscle actin) and FSP-1 positive. Isolated MAF were not contaminated with melanoma cells as they were negative for melanoma cells specific markers (melanoma cocktail, ab733, Abcam, Cambridge, MA) (Figs 1F and 2A). Human melanoma cells (C8161 [16]) were used as positive control for melanoma cells specific markers (melanoma cocktail). Both MAFs and normal human skin fibroblasts FF2441 were cultured in identical conditions. Expression of 84 genes involved in Notch signaling pathway was quantitatively analyzed by *RT² Profiler™ PCR Array*. Gene expression of *Notch1*, *Notch4*, *Delta-like-1* (*Dll1*), *Hes-1* and *Hey-1* was significantly down-regulated (fold change cut-offs of >2) in MAFs (Fig 2B) compared with normal human skin fibroblasts. The decreased protein expression of activated Notch1, full-length Notch4, Dll1, Hes-1 and Hey-1 in MAFs was validated by immunoblot (Fig 2C). Blots were cropped from different gels. Uncropped images of immunoblot are available in *S1 File*. Similarly, we found that the Notch pathway activity, as evidenced by elevated levels of Notch1 and Hes-1, was also higher in NHDF than MAF (Fig 2D). These results indicate that the Notch pathway activity is high in both neonatal and adult normal skin fibroblasts. Collectively, our data demonstrated that Notch pathway activity is low in MAFs relative to normal human skin fibroblasts, indicating that the intracellular Notch pathway is inhibited in MAFs.

### Activation of Notch1 pathway decreases MAF cell viability and proliferation

**Fig 3:**
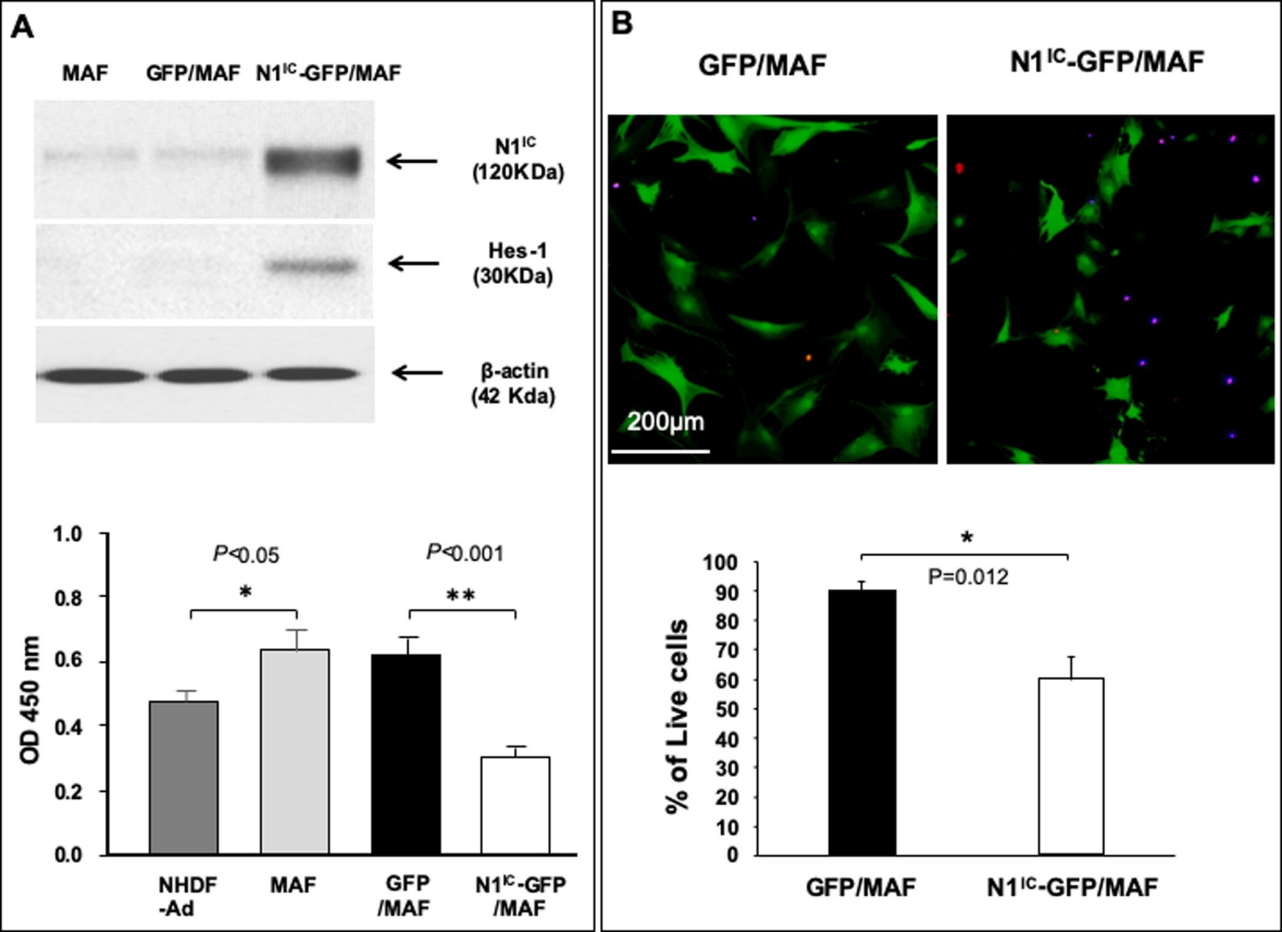
The Notch1 activation decrease MAF cell viability and proliferation. **A.** *Top*: overexpression of N1^IC^ in N1^IC^-GFP/MAF detected by immunoblot. increased Hes-1 expression demonstrates that the Notch pathway is activated. β-actin was used as loading control. *Bottom*: high intracellular Notch1 activity inhibits MAF proliferation *in vitro* as determined by WST cell proliferation assay. **B.** High activity of the intracellular Notch1 pathway decreases MAF cell viability. *Top*: images of cells in co-staining assay. Dead cells were double-positive [TUNEL (Red)^+^ and active caspase-3 (Blue)^+^], indicating that they are apoptotic cells. A single scale bar in a panel of pictures is representative for all pictures. *Bottom*: % of live cells. All data are presented as mean ± SD based on three independent experiments.

The above findings lead us to ask whether alteration of intracellular Notch pathway activity affects cell biology of MAFs. The Notch1 pathway was constitutively activated by transduction of a mixture of three MAF lines (1:1:1) with N1^IC^-GFP/lentivirus. The purpose of using cell mixtures of three MAF lines at 1:1:1 ratio is to avoid possible variation of individual MAF line and to test the general effect of MAFs engineered to carry high Notch pathway activity on melanoma cell growth. Three MAFs exhibited comparable growth rate (*S4 Fig*) and mixing them together won’t result in significant imbalance in the ratio of three lines of MAFs and selection of one MAF line from the mixture. GFP/lentivirus-transduced and un-transduced MAFs were used as controls. Normal human dermal fibroblasts from adults (NHDF-Ad) were also included as controls. The cells were named as N1^IC^-GFP/MAF, GFP/MAF and MAF accordingly. N1^IC^-GFP/MAF and GFP/MAF were sorted by FACS based on GFP. Levels of GFP in N1^IC^-GFP/MAF were slightly lower than those in GFP/MAF, because the GFP gene was constructed farther away from the promoter that drives expression of N1^IC^-*ires*-GFP genes in the N1^IC^-GFP/lentiviral vector compared to GFP/lentiviral vector. Expression of N1^IC^ and activation Notch pathway as indicated by elevated Hes-1 expression in N1^IC^-GFP/MAF were confirmed by immunoblot (Fig 3A, *Top*). We observed that enforced activation of the Notch1 pathway significantly slowed MAF cell growth as determined by WST cell proliferation assay (Fig 3A, *Bottom*). Notch1 pathway activation also induced cell apoptosis of MAFs as detectable by co-staining cells with TUNEL (BrdU-Red) and active Caspase-3 (Alexa Fluor 405). As shown in Fig 3B, almost all TUNEL-positive cells are also active Caspase-3-positive (red and blue double-positive cells). See *S5 Fig* for individual color of immunofluorescence. These data demonstrated that increasing intracellular Notch1 activity decreases MAF cell viability and proliferation.

### Increasing Notch1 pathway activity reverses tumor regulatory phenotype of MAFs *in vitro*

**Fig 4:**
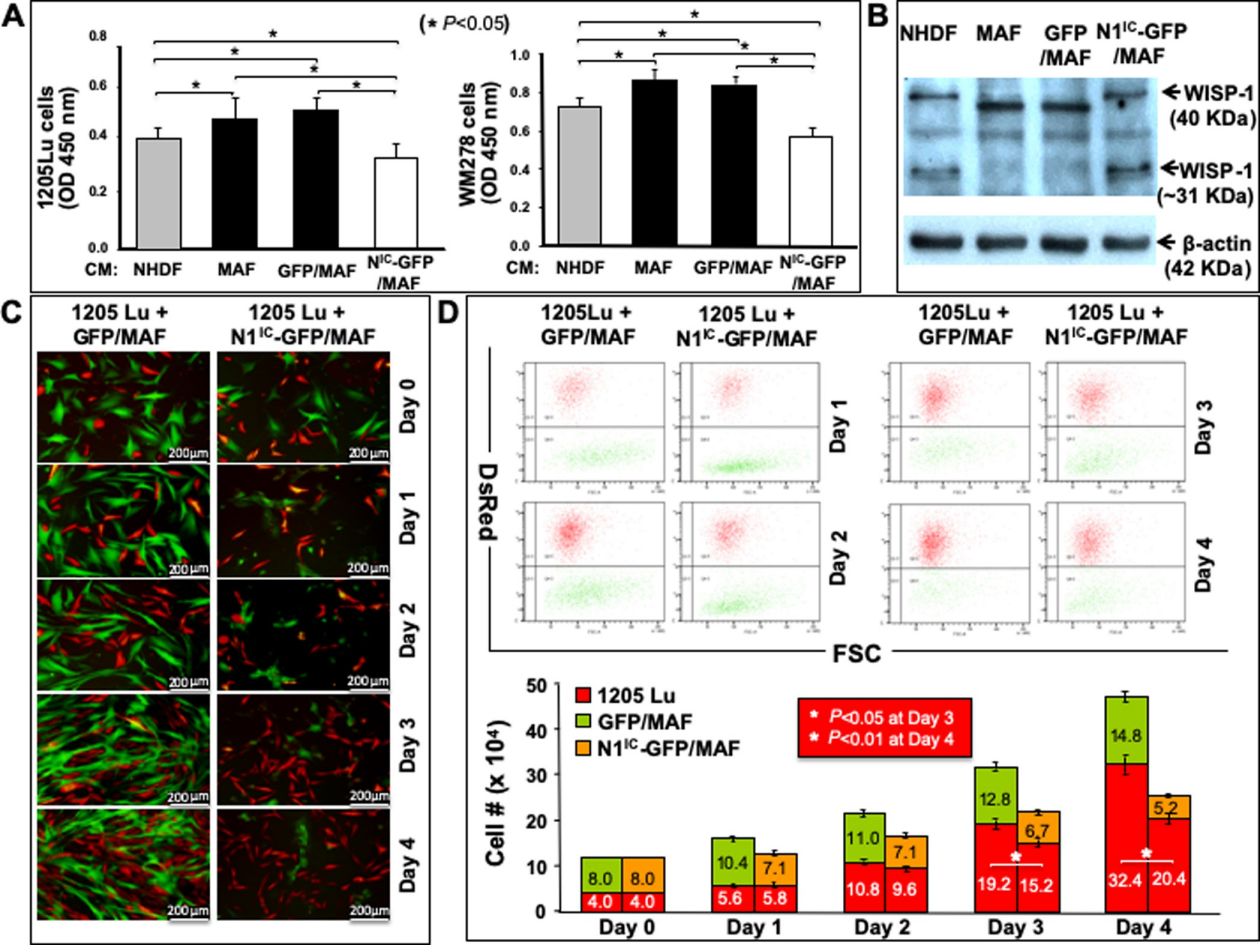
MAFs carrying high Notch1 activity slow melanoma cell growth in co-cultures. **A.** Effect of CM (conditioned medium) from NHDF, N1^IC^-GFP/MAF, GFP/MAF and MAF on cell growth of melanoma cells (1205Lu and WM278). OD values are shown. * *P*<0.05. **B.** Elevated expression of WISP-1 in N1^IC^-GFP/MAF detected by immunoblot. β-actin was used as loading control. **C.** Representative images of cell co-cultures on various days. **D.** Number of co-cultured DsRed^+^1205Lu melanoma cells counted by flow cytometer at different days. Numbers of melanoma cells and MAFs are calculated based on % of DsRed^+^ 1205Lu, GFP/MAF and N1^IC^-GFP/MAF measured by flow cytometry and total cell counts in each well of co-cultures. All data are presented as mean ± SD based on three independent experiments.

We further asked whether modification of intracellular Notch pathway activity in MAFs could alter their tumor regulatory role. The role of N1^IC^-GFP/MAF vs. GFP/MAF vs. MAF in regulating human melanoma cell growth was first tested by examination of the effect of conditioned medium (CM) collected from N1^IC^-GFP/MAF vs. GFP/MAF vs. MAF on melanoma cell growth. Growth of human melanoma cells derived from vertical growth phase (WM278) and metastatic phase (1205Lu) was tested by WST cell proliferation assay. We found that CM from N1^IC^-GFP/MAF significantly repressed melanoma cell growth (Fig 4A) compared to CM from GFP/MAF and MAF. To confirm the inhibitory effect of N1^IC^-GFP/MAF is caused by the Notch1 activation, we employed a dominant-negative mutant of Mastermind-like 1 (DN-MAML-1) to inhibit activated Notch pathway in N1^IC^-GFP/MAF, and then tested effect of the conditioned medium (CM) derived from DN-MAML-1/N1^IC^-GFP/MAF *vs*. Mock/N1^IC^-GFP/MAF on growth of melanoma cells. We found that compared to that of Mock/N1^IC^-GFP/MAF, CM from DN-MAML-1/N1^IC^-GFP/MAF could significantly relieve tumor-inhibitory effect of N1^IC^-GFP/MAF on melanoma cell growth (*S6 Fig*). These data confirmed that MAFs engineered with high Notch pathway activity indeed have a negative effect on the growth of melanoma cells. Above results suggested that certain soluble factors released by N1^IC^-GFP/MAF, either upregulated or downregulated by Notch pathway activation, might be responsible for decreased melanoma cell proliferation. We then examined the possible induction of WISP-1 (WNT1-inducible-signaling pathway protein-1) expression in N1^IC^-GFP/MAF, as we had previously found that Notch1 activation upregulates WISP-1 expression and WISP-1 is responsible for mediating inhibitory effect of stromal fibroblasts carrying high Notch1 pathway activity on melanoma growth [11]. Immunoblotting analysis showed that N1^IC^-GFP/MAF expressed elevated levels of WISP-1 compared to GFP/MAF and MAF (Fig 4B), suggesting that WISP-1 might mediate the observed inhibitory effect of N1^IC^-GFP/MAF on melanoma cell growth. Next, we tested the effect of N1^IC^-GFP/MAF vs. GFP/MAF on melanoma cell growth in MAF−melanoma cell co-cultures. N1^IC^-GFP/MAF or GFP/MAF were mixed with 1205Lu melanoma cells at a ratio of 2:1. Solely cultured melanoma cells were used as baseline control. Melanoma cell growth was significantly inhibited when co-cultured with N1^IC^-GFP/MAF vs. GFP/MAF. Representative images of cell co-cultures on various days were shown in Fig 4C. At day 1–4, portions of co-cultured cells were harvested for flow cytometry analysis to determine the numbers of melanoma cells (Red) and MAFs (Green). Cell numbers of both melanoma cells and MAF are shown, respectively, in Fig 4D. Melanoma cell growth was significantly inhibited when co-cultured with N1^IC^-GFP/MAF vs. GFP/MAF. Consistent with Fig 3, the numbers of N1^IC^-GFP/MAF were also gradually decreased with the time in the co-culture. These results demonstrated that activation of the Notch1 pathway could reverse the tumor-regulating phenotype of MAFs from tumor-promoting to tumor-suppressing.

### Increasing Notch1 pathway activity in MAFs inhibited melanoma growth *in vivo*

**Fig 5:**
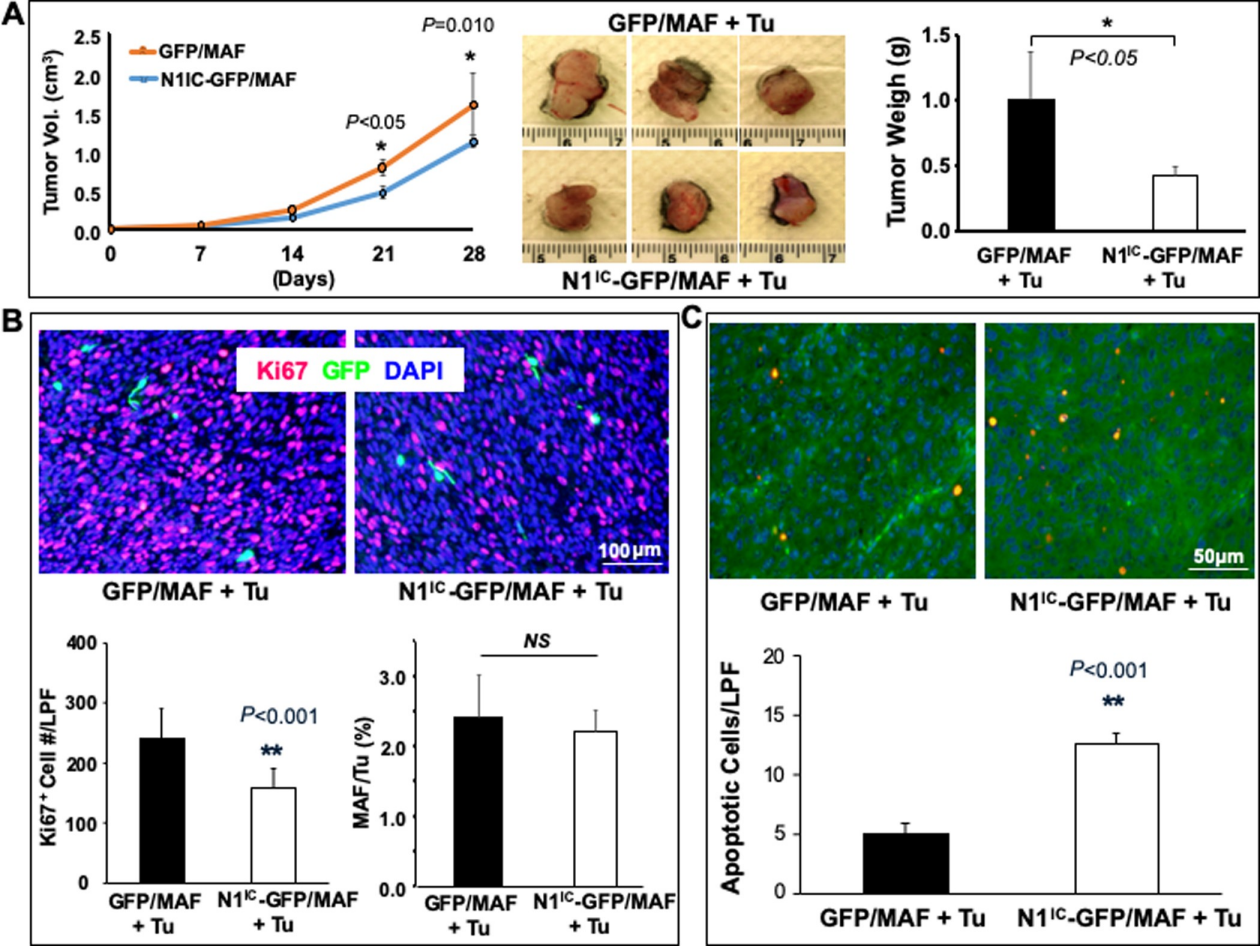
Increasing Notch1 activity in MAFs inhibits melanoma growth *in vivo*. **A.** *left*: tumor growth curve. *middle*: three representative images of resected tumors from each group [Tu stands for tumor cells (1205Lu)]. *right*: Tumor weight measured right after resection 4 weeks post co-grafting (n = 6 tumors/group). **B.** Immunostaining of tumor tissues. Fewer Ki67^+^ cells are detectable in 1205Lu co-grafted with N1^IC^-GFP/MAF compared to that co-grafted with GFP/MAF. *Top*: representative images; *Bottom*: number of Ki67^+^ cells per low power field (LPF, 10X) and % of N1^IC^-GFP/MAF vs. GFP/MAF in resected tumor tissues per LPF. **C.** More apoptotic tumor cells were detectable in 1205Lu co-grafted with N1^IC^-GFP/MAF vs. GFP/MAF. *Top*: representative images. Apoptotic tumor cells were co-stained with TUNEL (Red) and Luc (Green) and double-positive cells (orange color); *Bottom*: number of apoptotic cells per low power field (LPF, 10X). All data are mean ± SD based on 5 random sections per tumor tissue, n = 6 tumors/group.

Next, we conducted a co-grafting experiment by co-implanting MAFs and human melanoma cells on mouse skin. Mixtures of 2.4 x 10^6^ cells (at ratio of 2:1 of MAF: melanoma cells) of N1^IC^-GFP/MAF + 1205Lu vs. GFP/MAF + 1205Lu were co-grafted intradermally on dorsal skin of SCID mice (n = 6/group). Tumor size was recorded by weekly measurement of tumor length and width using a caliper and average tumor volumes were plotted in Fig 5A, *left*. Mice were sacrificed 4 weeks post co-grafting and skin tumors were resected. The resected tumors co-grafted with N1^IC^-GFP/MAF were smaller and lighter than that co-grafted with GFP/MAF (Fig 5A, *middle* and *right*). Grafted tumors were encapsulated, and no local invasion or distal tumor metastasis was detectable at the time of tumor resection. Melanoma growth was significantly inhibited in the presence of N1^IC^-GFP/MAF compared to that co-grafted with GFP/MAF in week 4 (Fig 5A, *right*). Immunostaining revealed a significantly lower number of Ki67^+^ cells in tumors co-grafted with N1^IC^-GFP/MAF (Fig 5B). Most of the proliferating cells (Ki67^+^) were GFP^-^ and were likely melanoma cells. Four weeks after co-grafting, tumor cells were present in overwhelming numbers, while co-grafted MAFs, which were stained as GFP^+^ cells, accounted for only ~2% of cells in the tumor tissues. There was no significant difference in the percent of GFP/MAF vs. N1^IC^-GFP/MAF (Fig 5B). It may be due to two reasons: (1) tumor cells grew quickly and became overwhelming, resulting in a small fraction of GFP/MAF *vs*. N1^IC^-GFP/MAF in xenografts. It is difficult to see accurate difference for such a small fraction of cells in tumor sections; (2) although N1^IC^-GFP/MAF grew slowly *in vitro*, but they might still alive within 4 week period of time *in vivo* (N1^IC^-GFP/MAF can remain in G0/G1 phase for certain period of time). Moreover, more apoptotic melanoma cells were detected in tumor tissues co-grafted with N1^IC^-GFP/MAF than those co-grafted with GFP/MAF by co-staining tumor sections with TUNEL (red) *and* anti-Luciferase (green, tumor cells are Luc^+^). Orange cells are double-positive cells (Fig 5C). Images showing individual fluorescence are shown in *S7 Fig*. These data demonstrated that increasing intracellular Notch1 pathway activity in MAFs could inhibit melanoma growth *in vivo*.

### Increasing Notch1 pathway activity in MAF attenuated tumor angiogenesis *in vivo*

**Fig 6:**
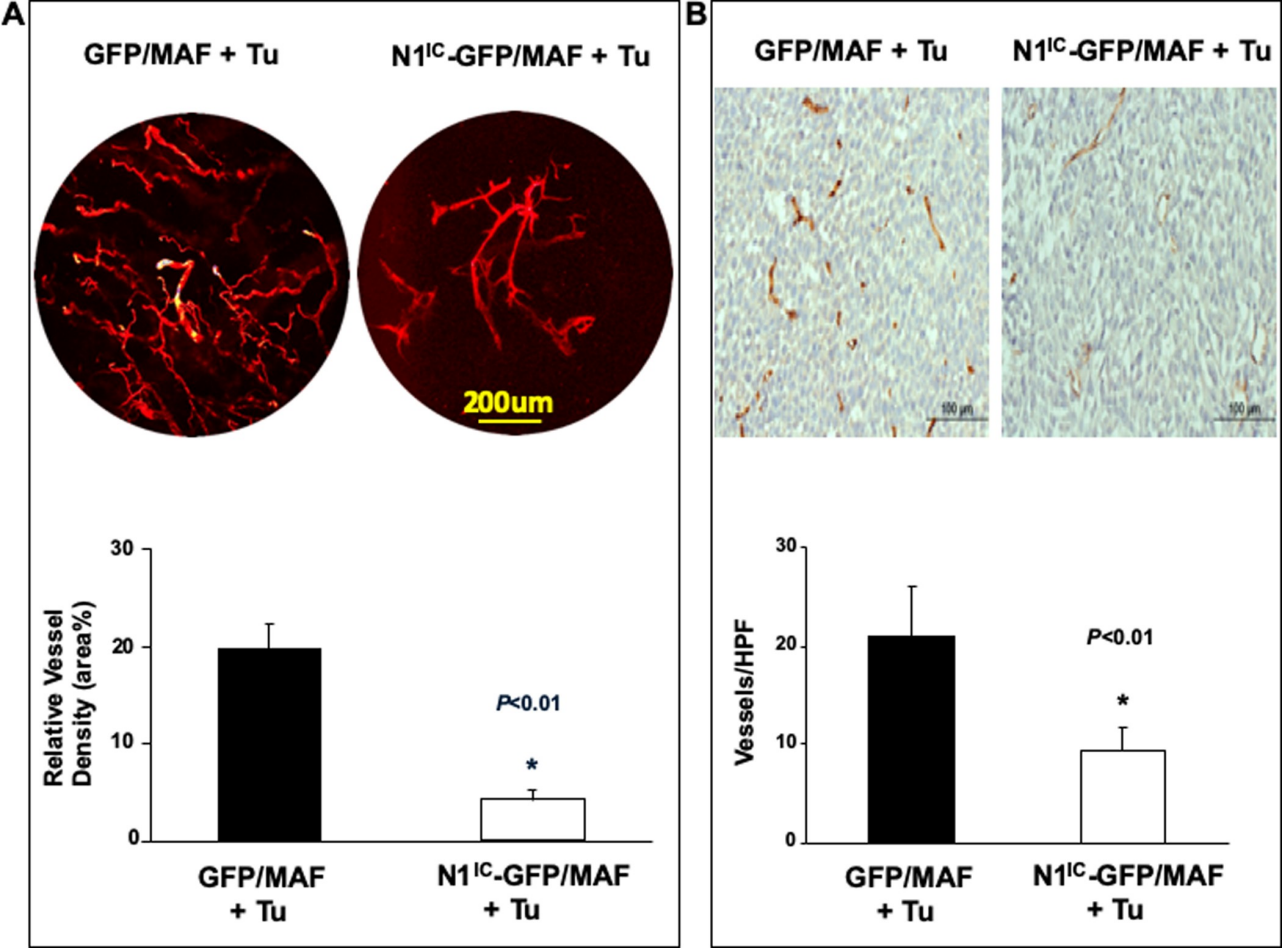
Increasing Notch1 activity in MAFs suppresses tumor angiogenesis. **A.** DiI-stained blood vessels in tumor tissue (Tu stands for tumor cells). *Top*: representative images; *Bottom*: relative blood vessel density calculated as % of red pixel in total area examined by confocal microscopy. Data are mean ± SD, n = 5 tumors/group. **B.** Significantly decreased blood vessel density is observed in 1205Lu co-grafted with N1^IC^-GFP/MAF. *Top*: representative images; *Bottom*: number of CD31^+^ vessels per LPF (10X). Data are mean ± SD based on 5 random sections per tumor tissue, n = 6 tumors/group.

In the tumor microenvironment (TME), CAFs interact not only with tumor cells but also with other stroma components, such as the vasculature. To examine the effect of increasing intracellular Notch pathway activity in MAFs on tumor angiogenesis, we evaluated blood vessel density by live animal whole-body Dil perfusion and subsequent laser scanning confocal microscopy in tumors harvested from additional MAF-melanoma cells co-grafted in SCID mice (n = 5/group). Significantly lower blood vessel density was observed in tumors co-grafted with N^IC^-GFP/MAF than in those co-grafted with GFP/MAF (Fig 6A). In addition, tissue sections from resected tumors described above in Fig 5A were stained with anti-CD31 antibody. Consistently, significantly fewer blood vessels were detectable in tumors co-grafted with N1^IC^-GFP/MAF compared to those co-grafted with GFP/MAF (Fig 6B). Together, these data implicated that angiogenesis in tumors co-grafted with N^IC^-GFP/MAF was repressed.

## Discussion

The incidence and mortality rates of melanoma have continued to increase in the United States [19]. Treatment of metastatic melanoma presents a challenge due to its poor response to standard chemotherapy and radiation. The discovery of novel targeted and immune therapies has revolutionized melanoma treatment [20–26]. However, melanoma continues to have a poor prognosis. Therefore, effective therapy for melanoma remains a major unmet clinical need. Our study identifies the Notch signaling pathway as a critical molecular determinant governing the tumor-regulating role of CAFs through therapeutic activation of the Notch pathway by targeting the TME to convert and reprogram CAFs from “tumor promoters” to “tumor suppressors”. This novel approach can lead to innovations in integrative cancer care with the development of novel adjuvant/neoadjuvant melanoma therapies.

The notch pathway in CAFs has several means of activation, such as CRISPR/Cas9, to introduce N1IC, or gene therapy approach or applying a Notch pathway activating compound. The Notch pathway activating compound can be identified though a high-throughput screening method and specifically activates CAF’s notch signaling pathway [27]. However, high Notch activity is oncogenic to melanoma [15, 28, 29] and Notch signaling functionality is cell context-dependent [30]. The key for precision medicine will be the development and clinical application of Notch-targeting agents that selectively increase Notch pathway activity in MAFs without increasing Notch pathway activity in melanoma cells. An alternative is to target the mechanism responsible for mediating the Notch-induced tumor-suppressing phenotype of MAFs through the utilization of the Notch pathway downstream targets, for example, WISP-1 [9, 11, 31]. Since WISP-1 is a soluble molecule and is thus easy to be directly administered, our previous findings provide a practicable agent to control melanoma progression using WISP-1 as a functional mediator of Notch1 signaling. In the current study, our data further support existence of such a Notch−WISP-1 cascade in MAFs, as we observed that MAFs carrying high Notch pathway activity express elevated levels of WISP-1. We have shown that the Notch-induced WISP-1 expression is Wnt 11-depedenent. There is a Notch-Wnt 11-WISP-1 axis in fibroblasts [9]. Potentially, WISP-1 can be an ideal and manageable therapeutic candidate to treat melanoma because melanoma cells barely express WISP-1 [11]. Moreover, WISP-1 appears to be responsible for mediating the Notch1-induced anti-tumor growth and anti-angiogenic effect of MAFs, as we previously demonstrated that attenuation of Notch1-upregulated WISP-1 expression by *WISP-1*-shRNA in fibroblasts could significantly liberate the inhibitory effect of N1^IC^-engineered stromal fibroblasts on tumor growth and angiogenesis in mouse model [11]. Future study is warranted to test biosafety and optimal dose, route, and frequency to administer WISP-1 in animal models.

Our current work focused on the assessment of the status of intracellular Notch signaling activity in MAFs and exploring role of the intracellular Notch signaling in determining tumor regulatory function of MAFs. It should be noted that the roles of Notch/ligand in mediating intercellular communication and roles of intracellular Notch signaling in MAFs or melanoma cells are two different topics. Whether Notch/ligand participate in cell-cell communication between fibroblasts−melanoma cells and/or fibroblast−endothelial cells remains unclear. It is also unclear how Notch signaling is differentially regulated in MAFs and melanoma cells in a single TME. Likely, the Notch pathway in different types of cells (MAFs, melanoma cells and endothelial cells) may be differentially regulated. Notch activation or inactivation may result in non-unified consequences in these cells and may employ different downstream targets and mechanisms. These questions are topics for future studies.

Although human metastatic melanoma cells (1205Lu) were tested in mouse experiments, no distant metastasis was detectable. This is not surprising, because it is well known that the incidence of spontaneous metastasis of grafted melanoma cells is very low in the murine melanoma model. It typically needs resection of the primary tumor in order for formation of distant metastases to occur [32]. Moreover, the 1205Lu cells may lose *in vivo* metastatic capability due to the long-term *in vitro* culture. Alternatively, it may be insufficient for a full course of metastasis to be completed within the time frame (4-weeks) of our experiments. Other aggressive metastatic melanoma cells may need to be tested over a longer time course in order to assess the effect of increasing Notch1 pathway activity in MAFs on melanoma metastasis.

How the Notch activity switches from a high status (ON) in normal fibroblasts to a low one (OFF) in MAFs remains a mystery and requests future investigation. Likely, the influence of the tumor and the TME results in a genetic or epigenetic facilitation of Notch1 inactivation. MAF’s Notch signaling pathway may be turned OFF by cell–cell interaction between MAFs and melanoma cells, or other tumor stromal cells, or by a yet unidentified inhibitory signaling cascade(s) initiated by cytokine(s) and ECM in tumor tissue. At physiological conditions, normal fibroblast partner cells switch due to of the invasion of melanoma cells and infiltration/recruitment of other tumor stromal cells. New communication or disruption of existing communication causing diminished Notch signaling in MAFs may be a result of this switch of partner cells. It is also unknown how tumor cells modulate stromal cells in the TME and whether there is a link between mutation types and status in melanoma cells and Notch activity in MAFs. Our findings warrant future study to elucidate mechanisms for down-regulated Notch activity in MAFs.

## Conclusions

Our study demonstrates that the Notch signaling is inhibited in MAFs. Increase of Notch pathway activity can reverse the tumor-regulating phenotype of MAFs from tumor-promoting to tumor-suppressing. Our study suggests that targeting melanoma by activating Notch1 signaling in MAFs may represent a novel therapeutic approach.

## Supporting information

S1 Fig Low Notch activity in MAFs. Individual color of 16 IF images of tissue microarrays stained with Alexa Fluor® 594-conjugated anti-Hes1 shown in Fig 1. A single scale bar in a panel of pictures is representative for all pictures.
(TIFF)

S2 Fig Low Notch activity in MAFs. Individual color of 16 IF images of tissue microarrays stained with FITC-conjugated anti-FSP-1 shown in Fig 1. A single scale bar in a panel of pictures is representative for all pictures.
(TIFF)

S3 Fig Low Notch activity in MAFs. Individual color of 16 IF images of tissue microarrays stained with DAPI shown in Fig 1. A single scale bar in a panel of pictures is representative for all pictures.
(TIFF)

S4 Fig Growth rate of three individual MAF cell lines *in vitro*. Cell growth was determined by WST cell proliferation assay. Three lines exhibit comparable growth rates. *N*.*S*.: not significant (ANOVA). Experiments were repeated three times.
(TIFF)

S5 Fig High activity of the intracellular Notch1 pathway decreases MAF cell viability. Individual color of IF images of co-stained N1^IC^-GFP/MAF and GFP-MAF as shown in Fig 3B. Images covering larger areas and more cells are shown in the top. A single scale bar in a panel of pictures is representative for all pictures.
(TIFF)

S6 Fig Reverse tumor-inhibitory function of N1^IC^-GFP/MAF by DN-MAML-1. The effects of conditioned medium (CM) derived from DN-MAML-1/N1^IC^-GFP/MAF *vs*. Mock/N1^IC^-GFP/MAF on growth of melanoma cells were determined by WST cell proliferation assay. CM from DN-MAML-1/N1^IC^-GFP/MAF could significantly relieve tumor-inhibitory effect of N1^IC^-GFP/MAF on melanoma cell growth. Experiments were repeated three times. * *P*<0.01.
(TIFF)

S7 Fig High Notch1 activity in MAFs induces cell apoptosis of tumor cells. Individual color of IF images of Fig 5C. A single scale bar in each panel of picture is representative for all pictures.
(TIFF)

S1 File (PDF)
