# Supplementary figures and images for: Converting melanoma-associated fibroblasts into a tumor-suppressive phenotype by increasing intracellular Notch1 pathway activity
Source: PLoS One. 2021 Mar 11;16(3):e0248260. doi: 10.1371/journal.pone.0248260 (PMC7951899; doi:10.1371/journal.pone.0248260)

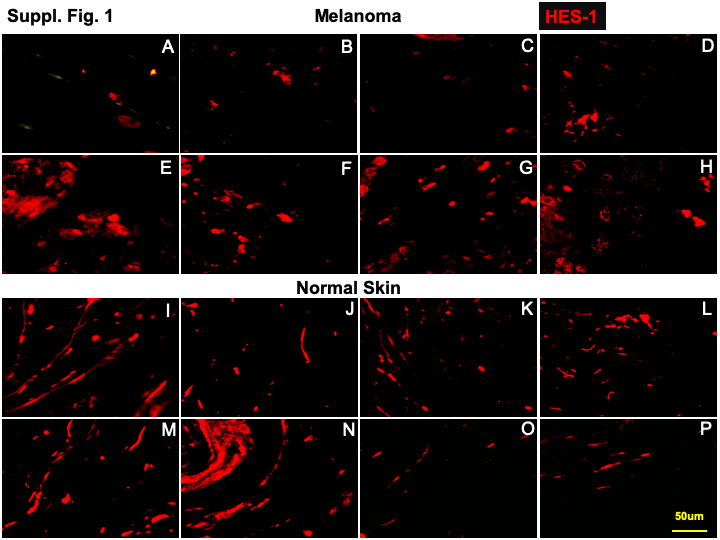

Supplement: S1 Fig — Individual color of 16 IF images of tissue microarrays stained with Alexa Fluor® 594-conjugated anti-Hes1 shown in Fig 1. A single scale bar in a panel of pictures is representative for all pictures. (TIFF) [file pone.0248260.s001.tiff]

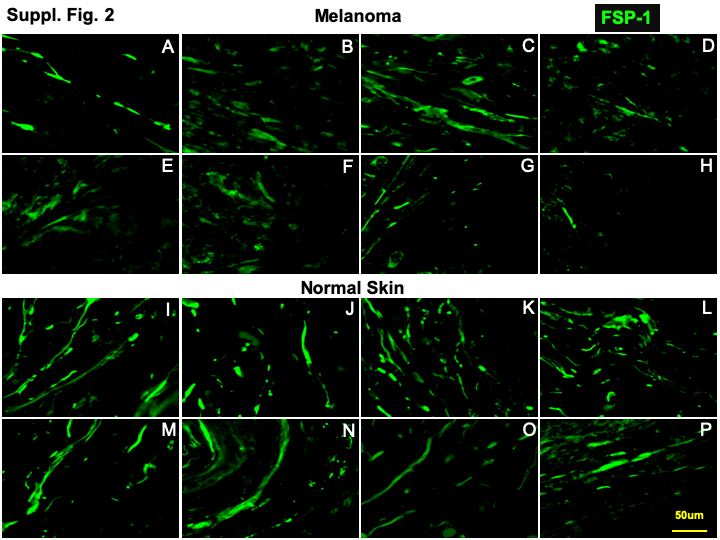

Supplement: S2 Fig — Individual color of 16 IF images of tissue microarrays stained with FITC-conjugated anti-FSP-1 shown in Fig 1. A single scale bar in a panel of pictures is representative for all pictures. (TIFF) [file pone.0248260.s002.tiff]

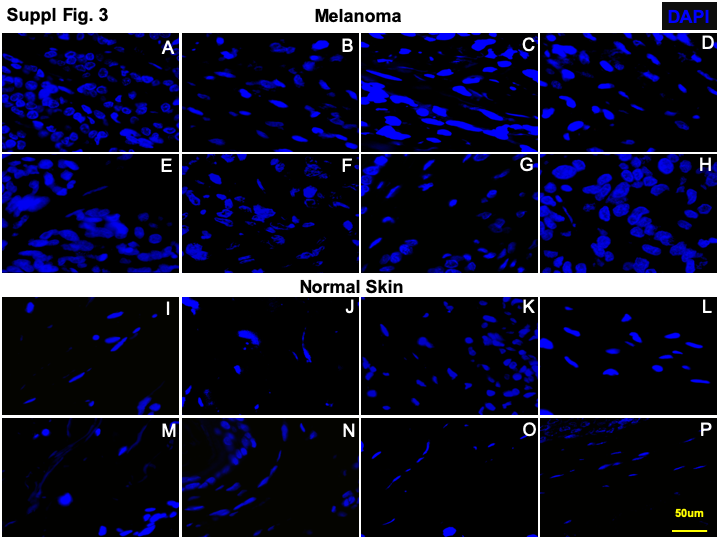

Supplement: S3 Fig — Individual color of 16 IF images of tissue microarrays stained with DAPI shown in Fig 1. A single scale bar in a panel of pictures is representative for all pictures. (TIFF) [file pone.0248260.s003.tiff]

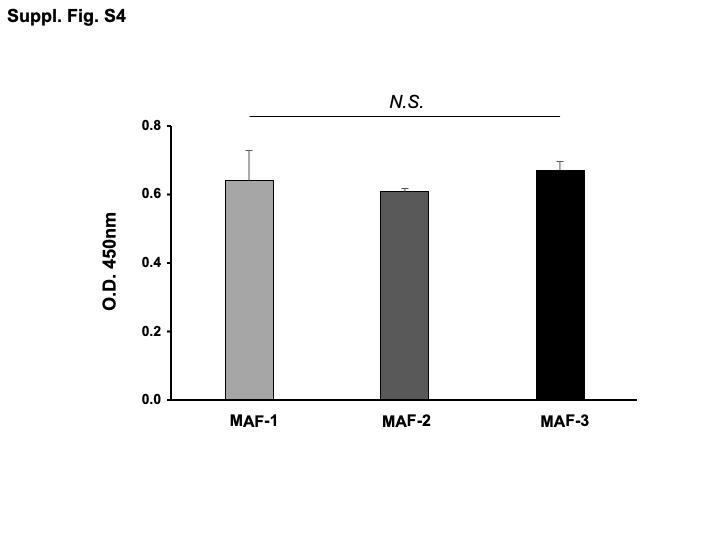

Supplement: S4 Fig — Cell growth was determined by WST cell proliferation assay. Three lines exhibit comparable growth rates. N.S.: not significant (ANOVA). Experiments were repeated three times. (TIFF) [file pone.0248260.s004.tiff]

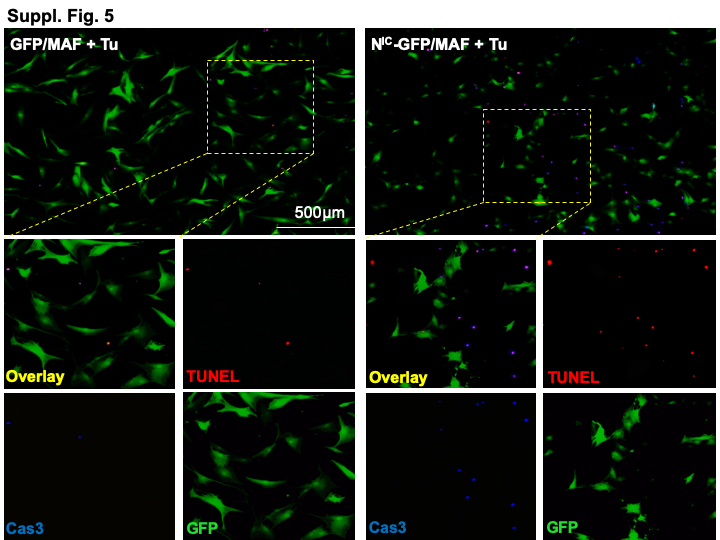

Supplement: S5 Fig — Individual color of IF images of co-stained N1IC-GFP/MAF and GFP-MAF as shown in Fig 3B. Images covering larger areas and more cells are shown in the top. A single scale bar in a panel of pictures is representative for all pictures. (TIFF) [file pone.0248260.s005.tiff]

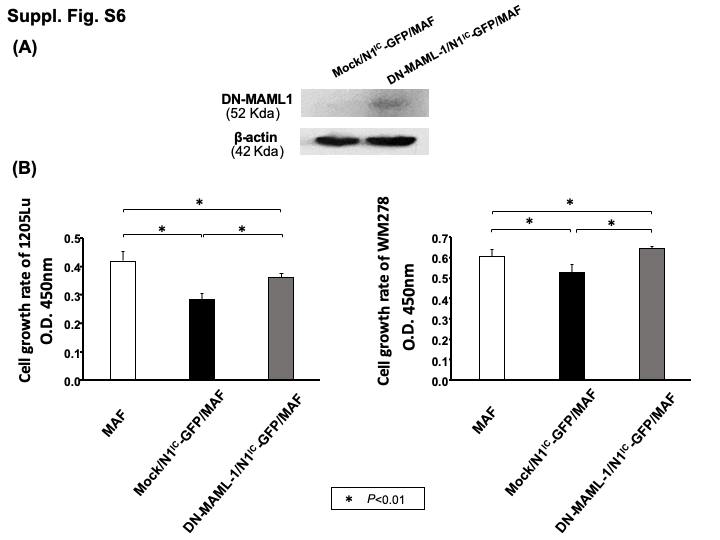

Supplement: S6 Fig — The effects of conditioned medium (CM) derived from DN-MAML-1/N1IC-GFP/MAF vs. Mock/N1IC-GFP/MAF on growth of melanoma cells were determined by WST cell proliferation assay. CM from DN-MAML-1/N1IC-GFP/MAF could significantly relieve tumor-inhibitory effect of N1IC-GFP/MAF on melanoma cell growth. Experiments were repeated three times. * P<0.01. (TIFF) [file pone.0248260.s006.tiff]

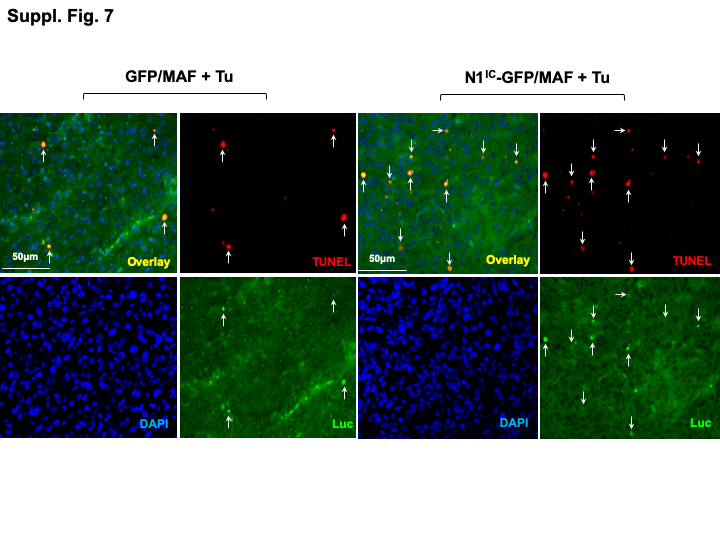

Supplement: S7 Fig — Individual color of IF images of Fig 5C. A single scale bar in each panel of picture is representative for all pictures. (TIFF) [file pone.0248260.s007.tiff]
