# Supplementary material for: Converting melanoma-associated fibroblasts into a tumor-suppressive phenotype by increasing intracellular Notch1 pathway activity
Source: PLoS One. 2021 Mar 11;16(3):e0248260. doi: 10.1371/journal.pone.0248260 (PMC7951899; doi:10.1371/journal.pone.0248260)

Fig. 2C Notch1 120 kDa

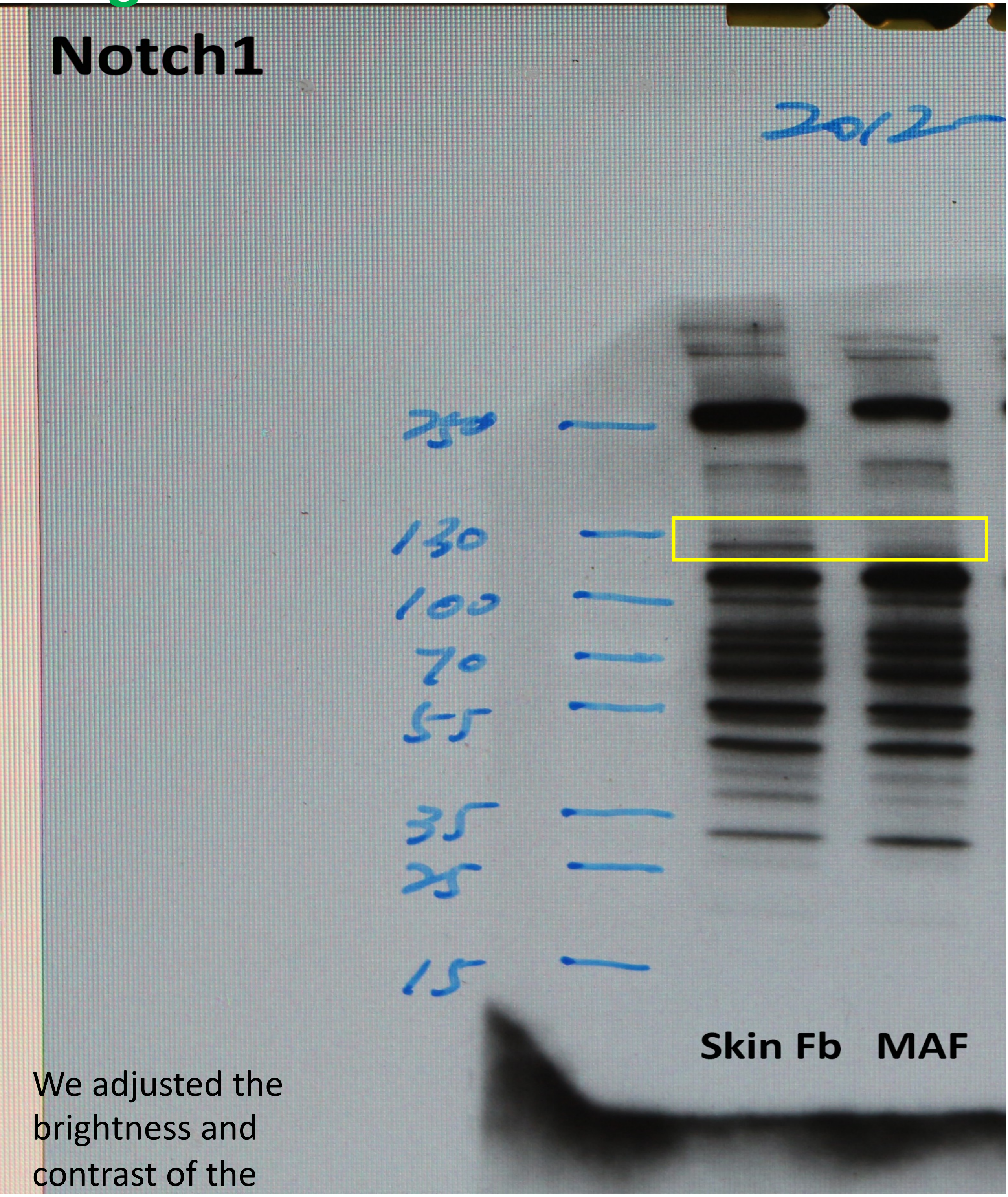

We adjusted the brightness and contrast of the original plot picture. Please find this small black dot in Fig2C Notch1

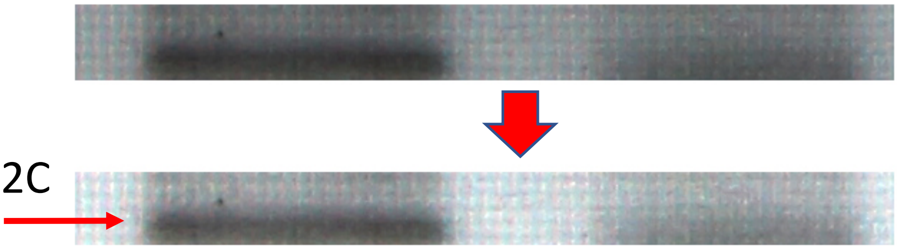

Fig. 2C Notch4 210kDa

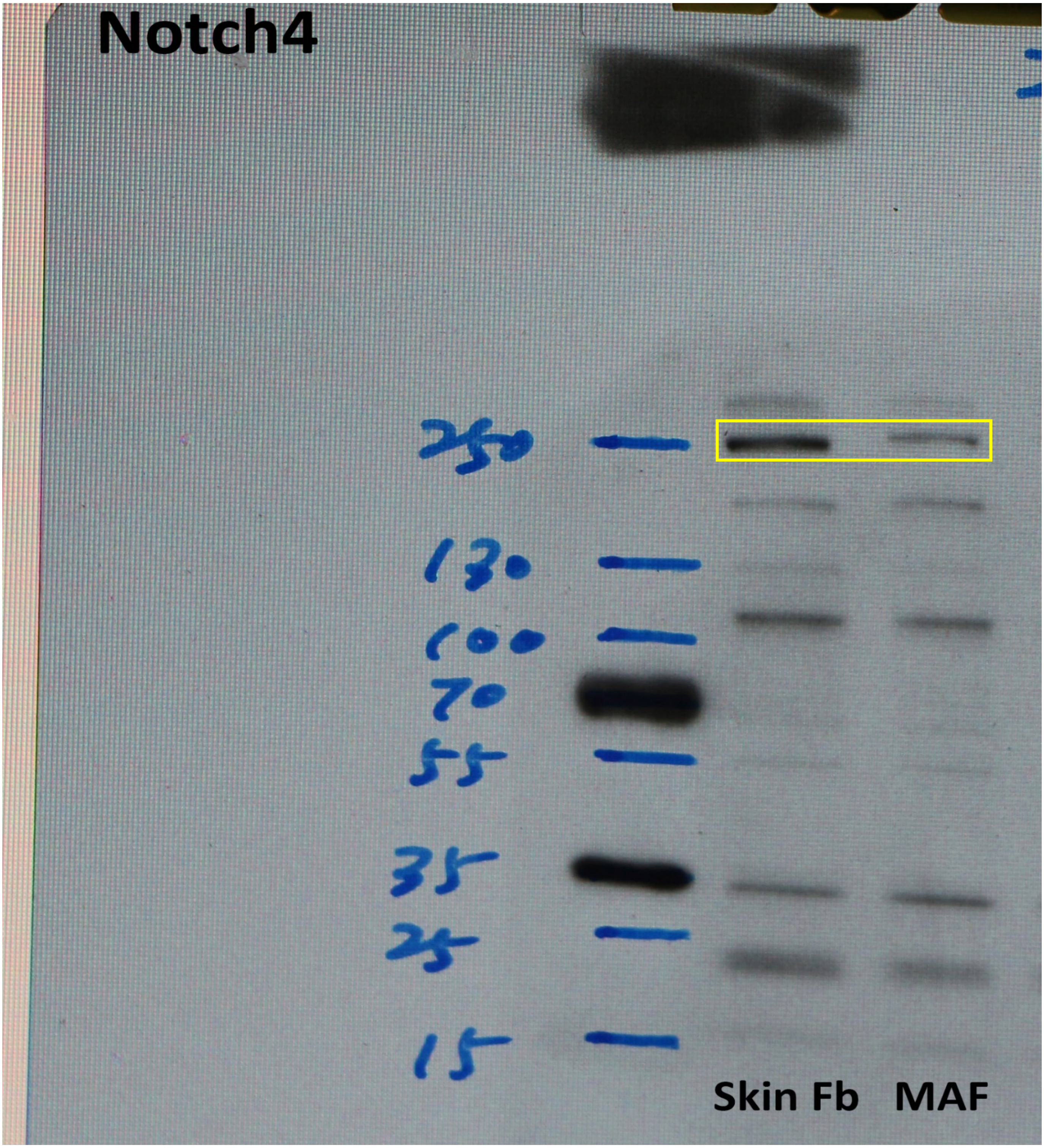

Fig. 2C DLL1 78kDa

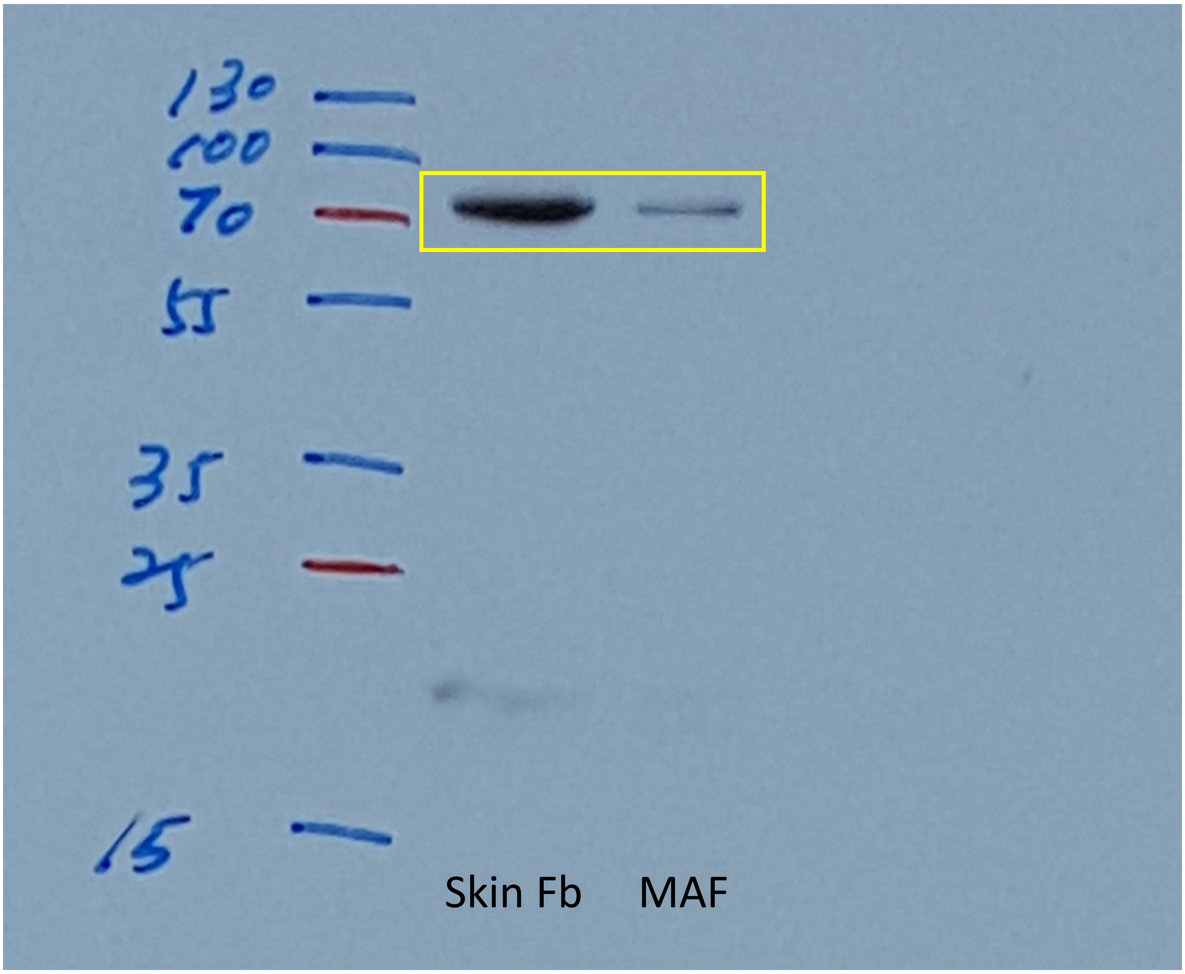

Fig. 2C Hes1 30kDa

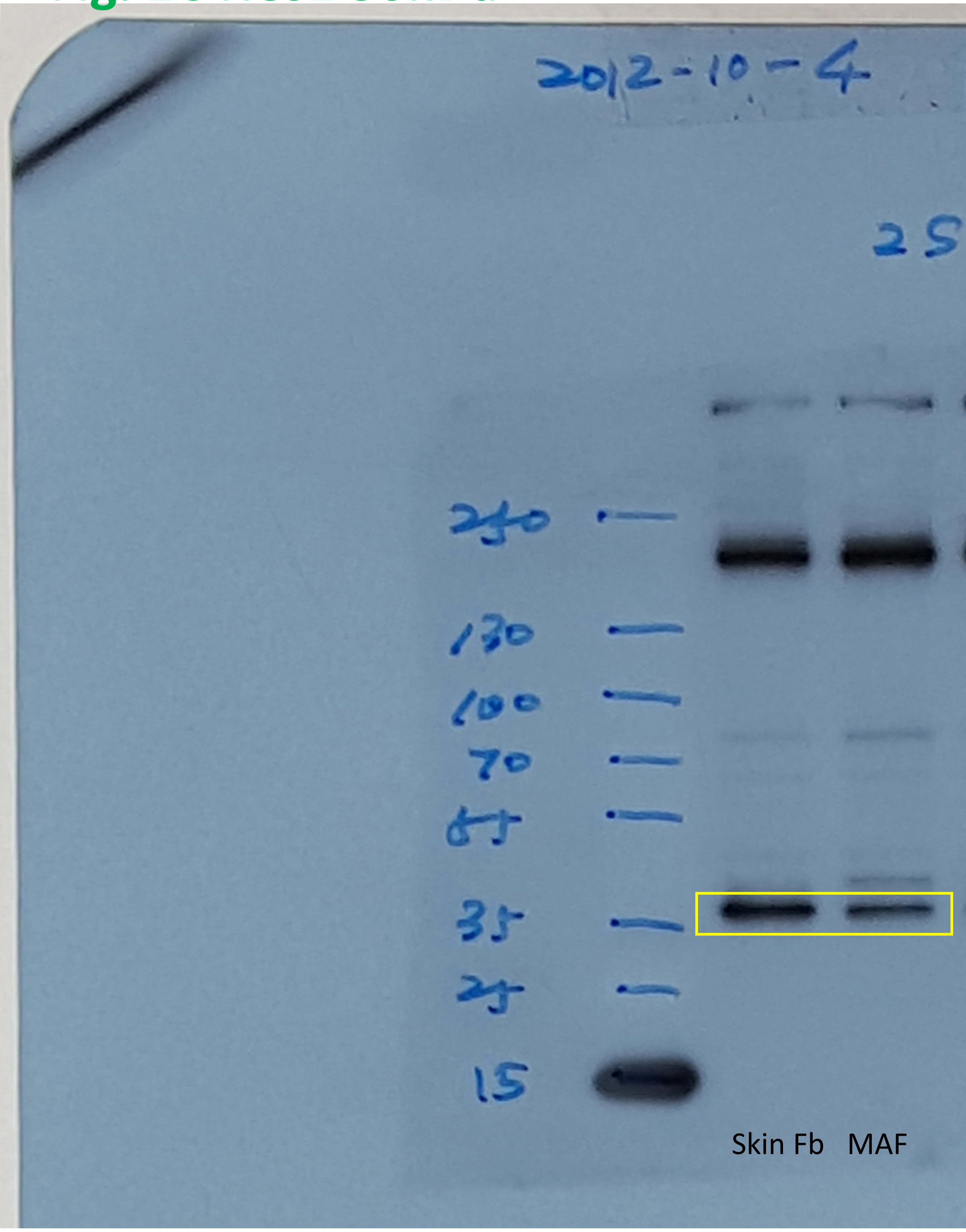

Fig. 2C Hey1 33kDa

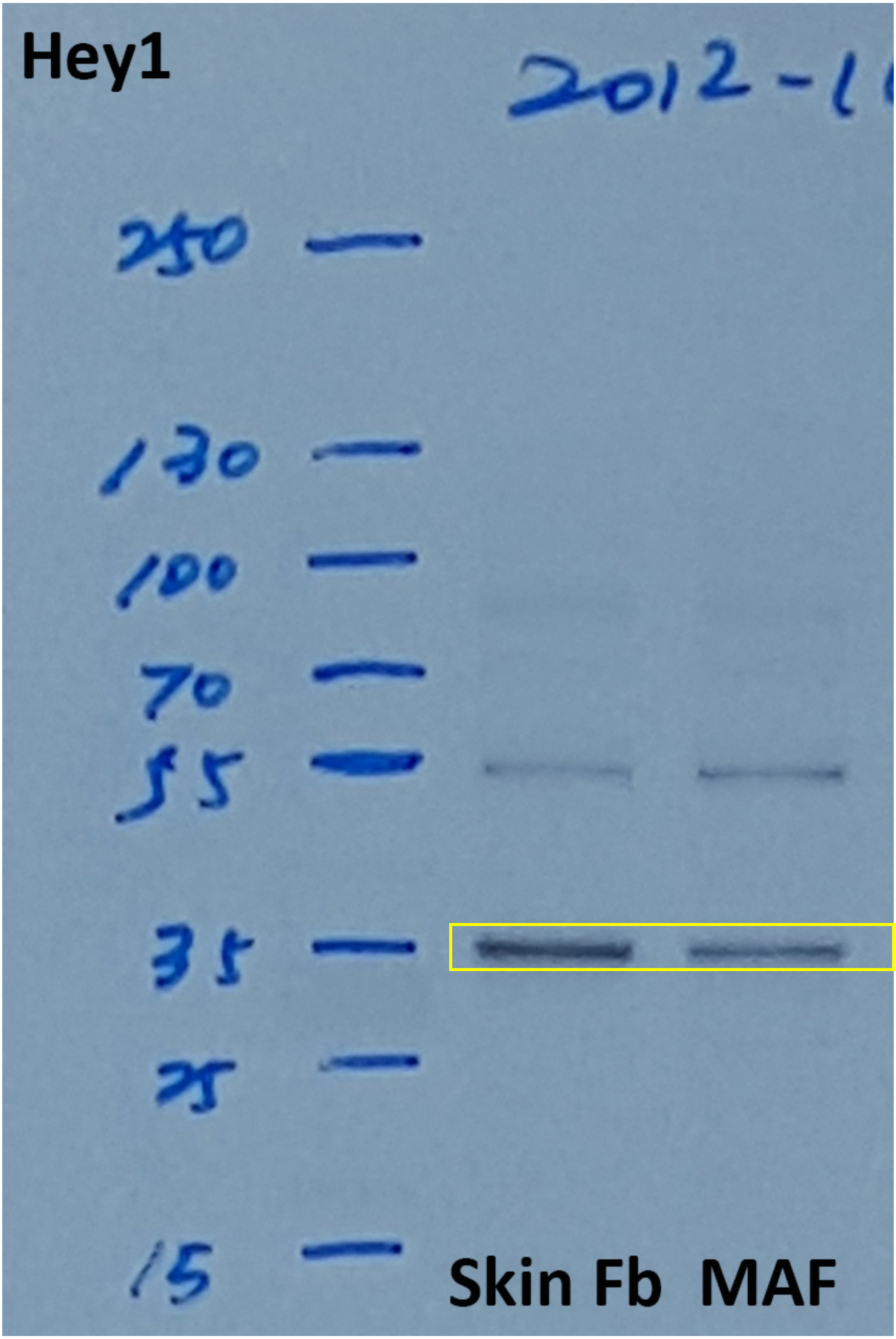

Fig. 2C β–actin 42kDa

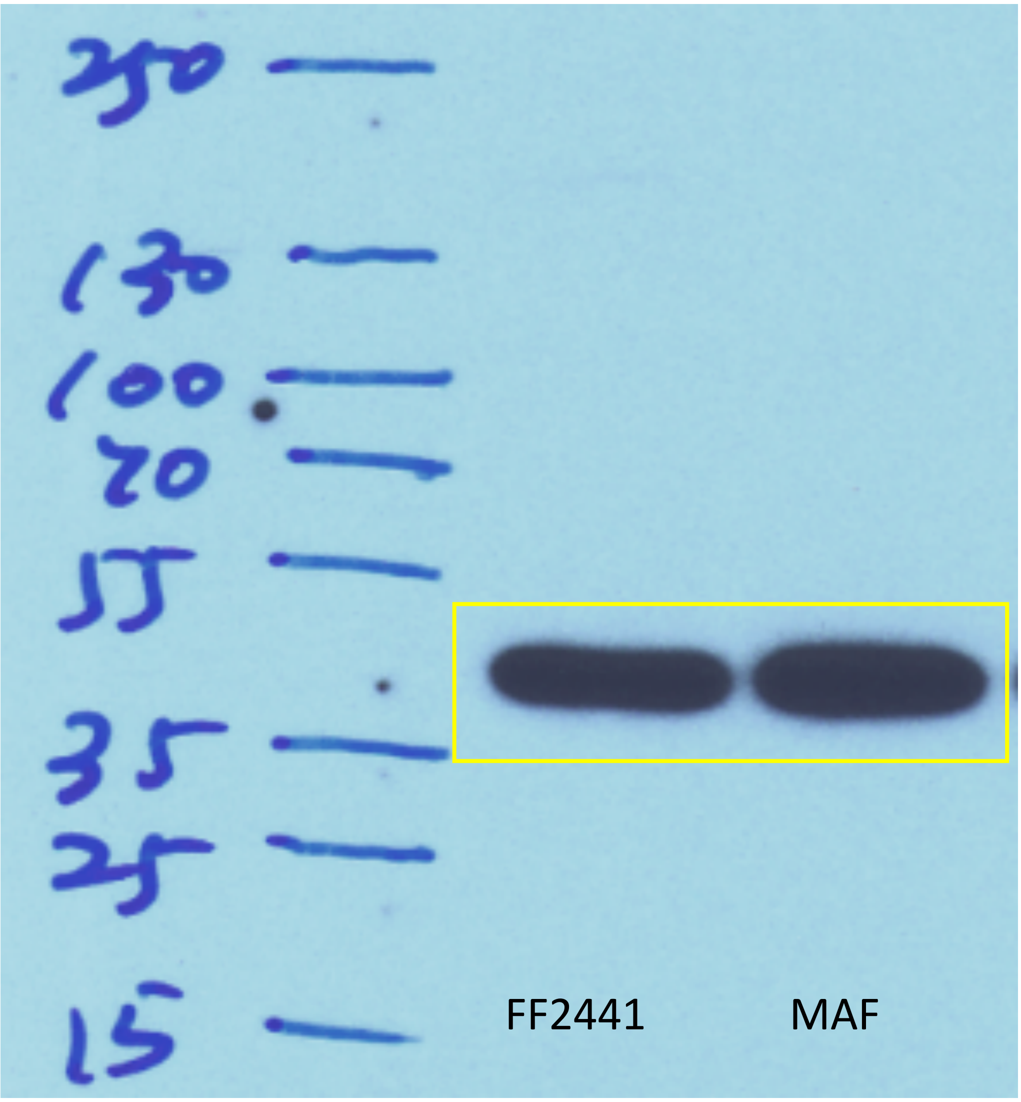

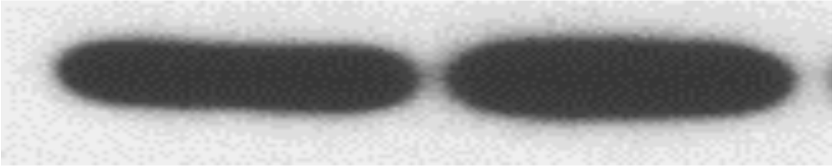

Cropped and deformed to fit space.

Fig. 2D Notch1 120kDa

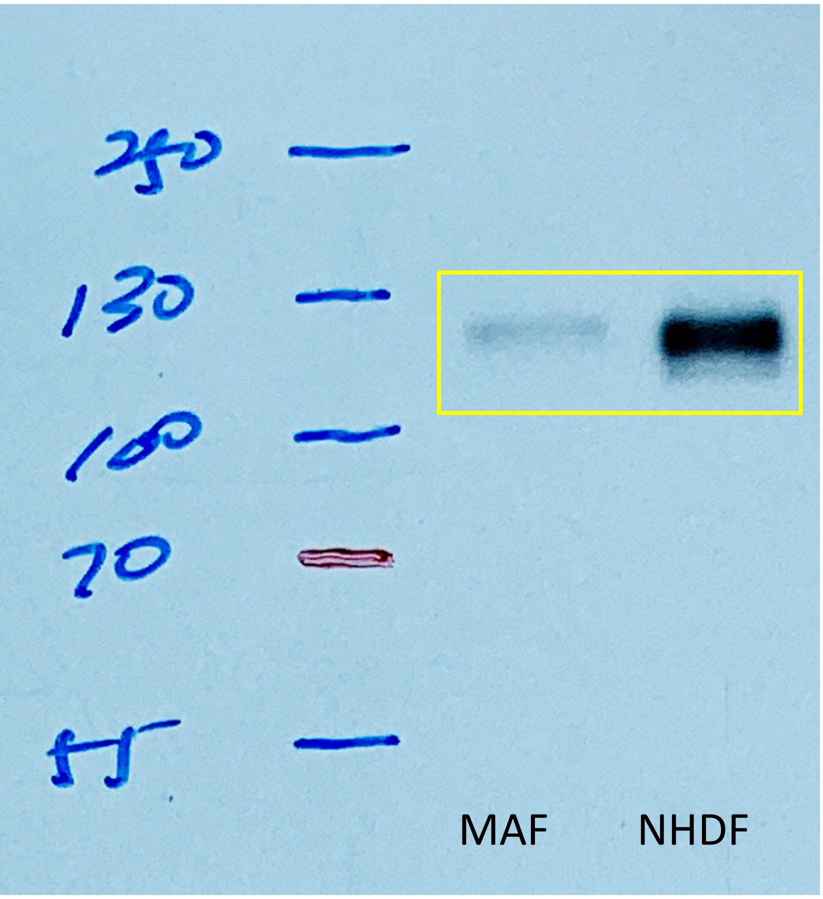

Cropped

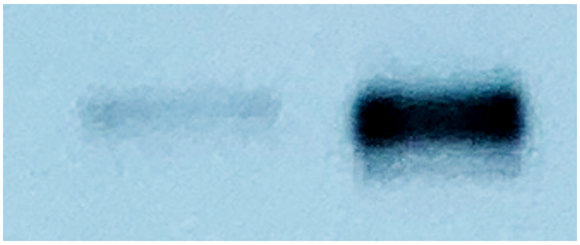

Flipped

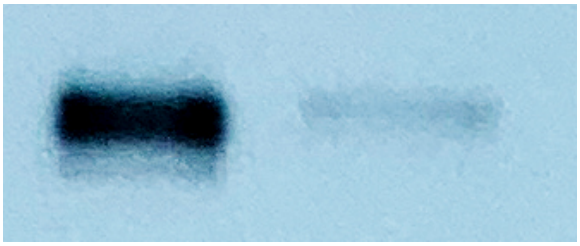

Fig. 2D Hes1 30kDa

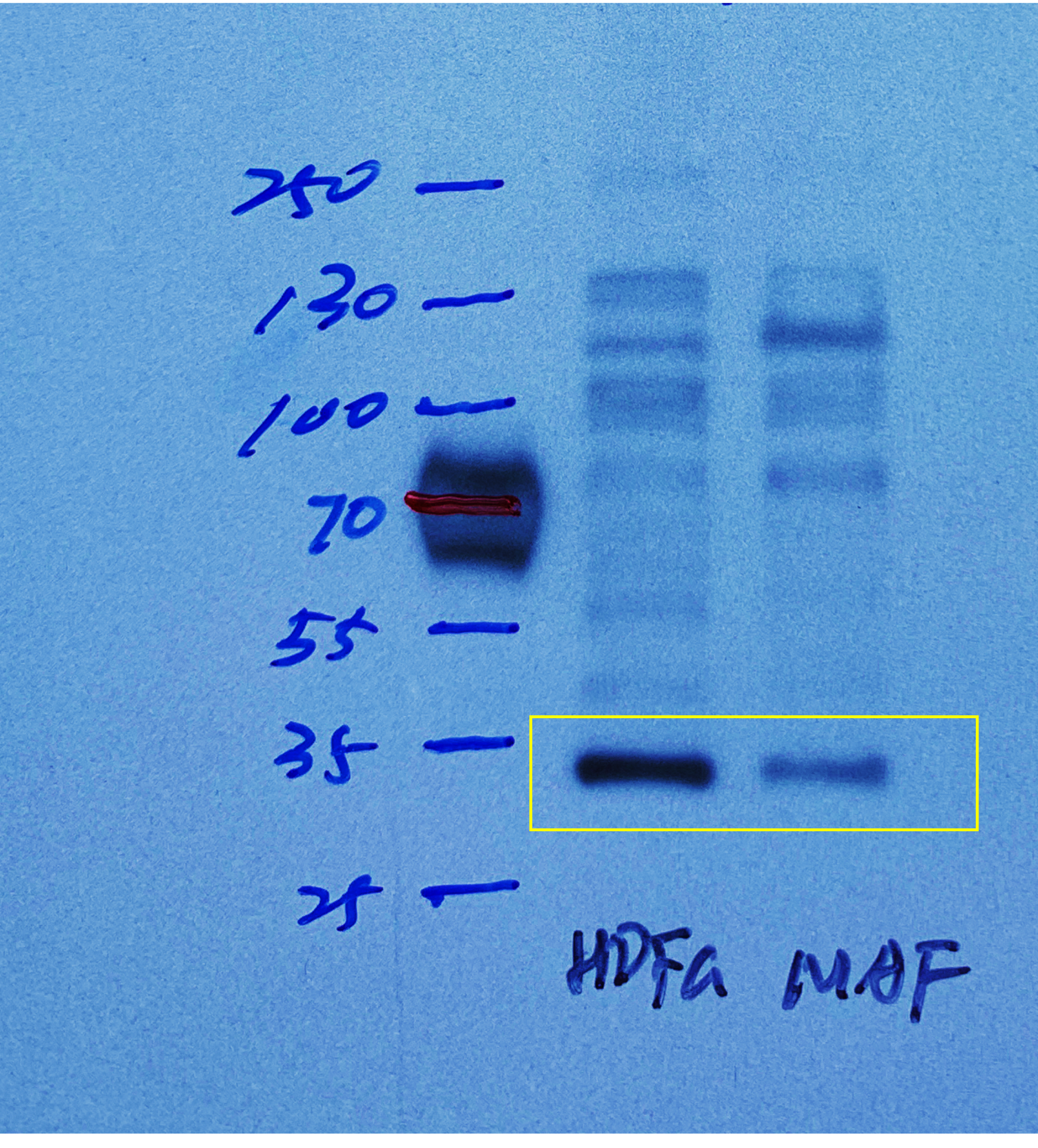

Fig. 2D β–actin 42kDa

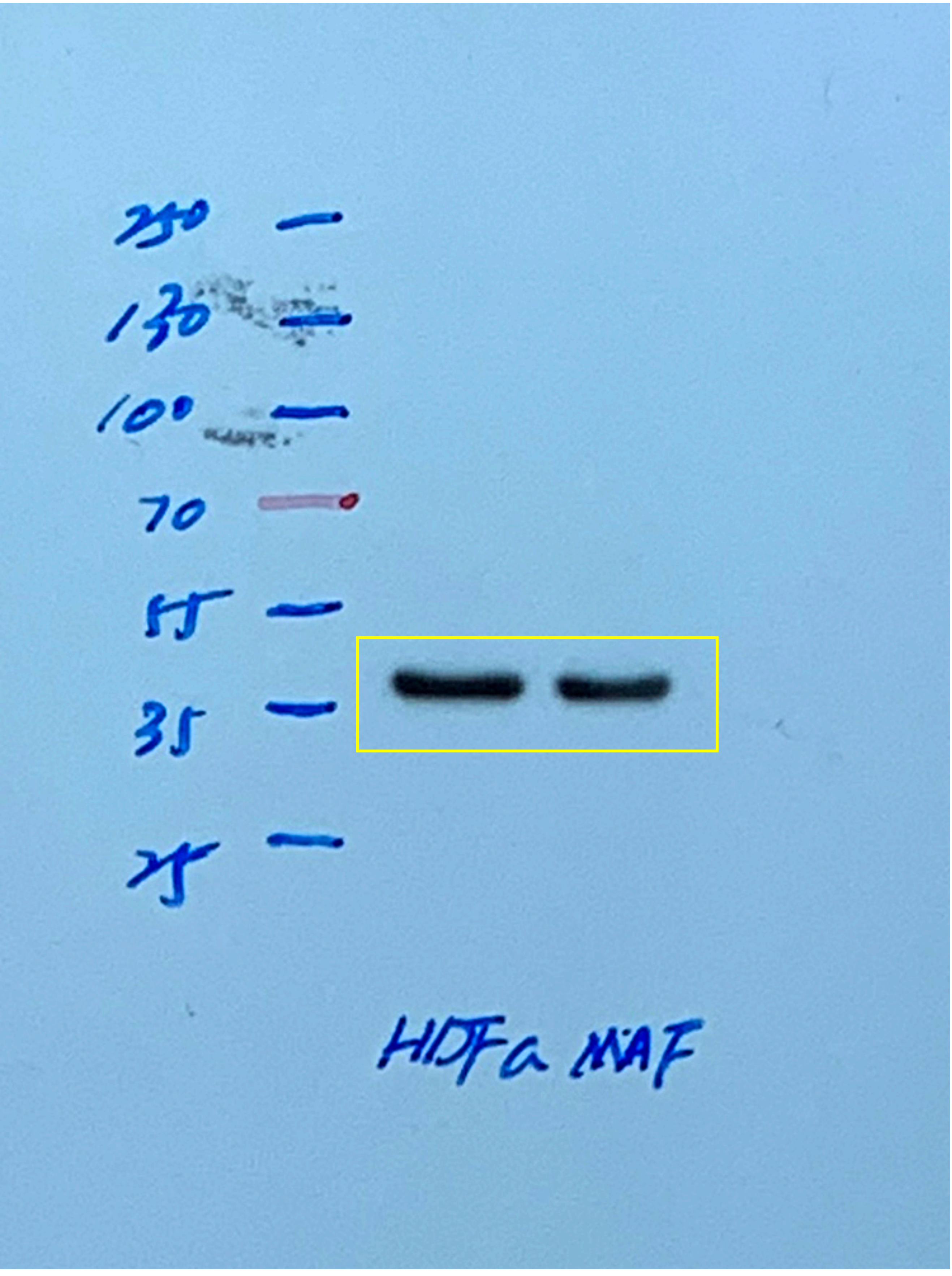

Fig3A Notch1 120kDa

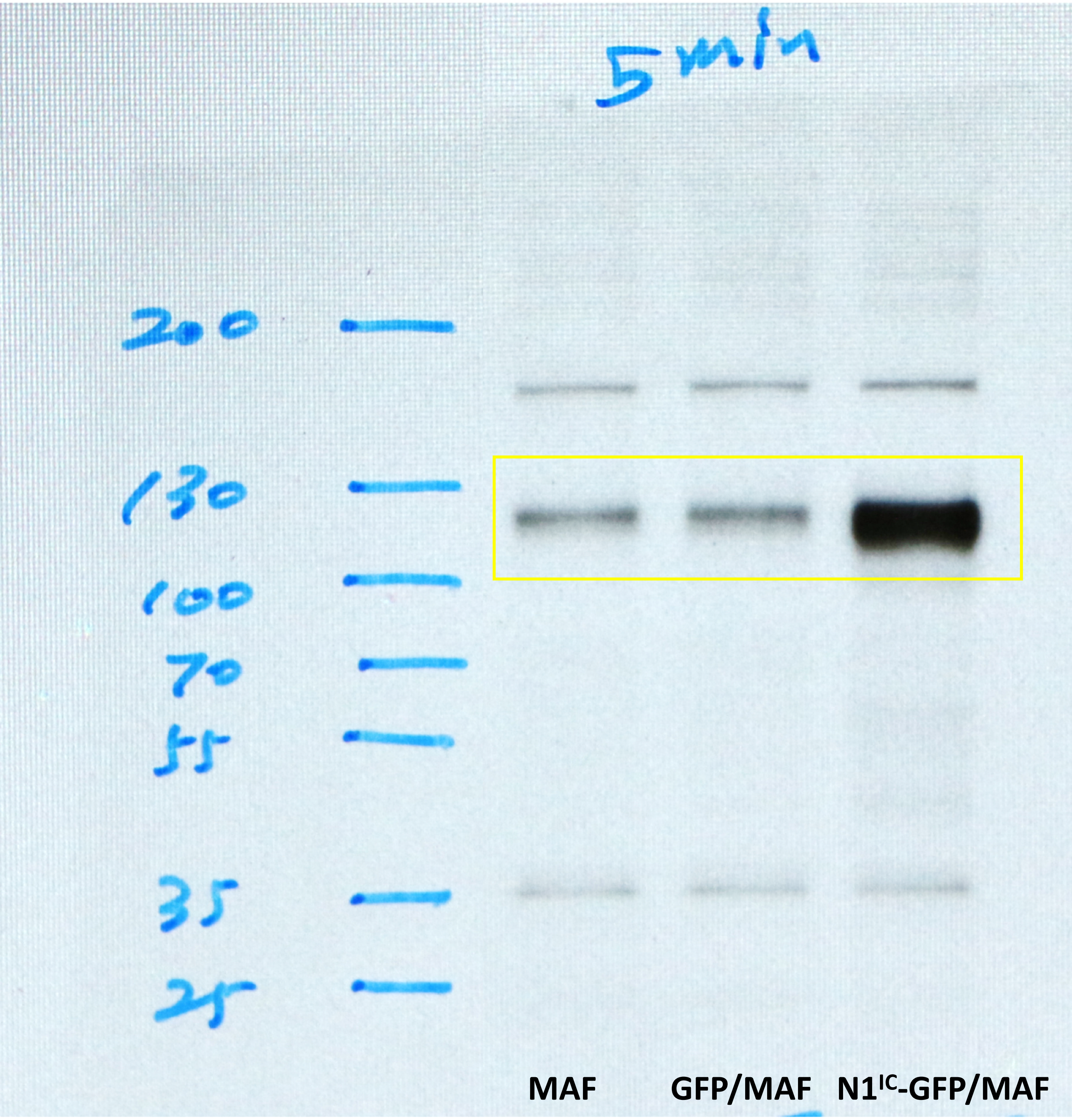

Fig3A Hes1 30kDa

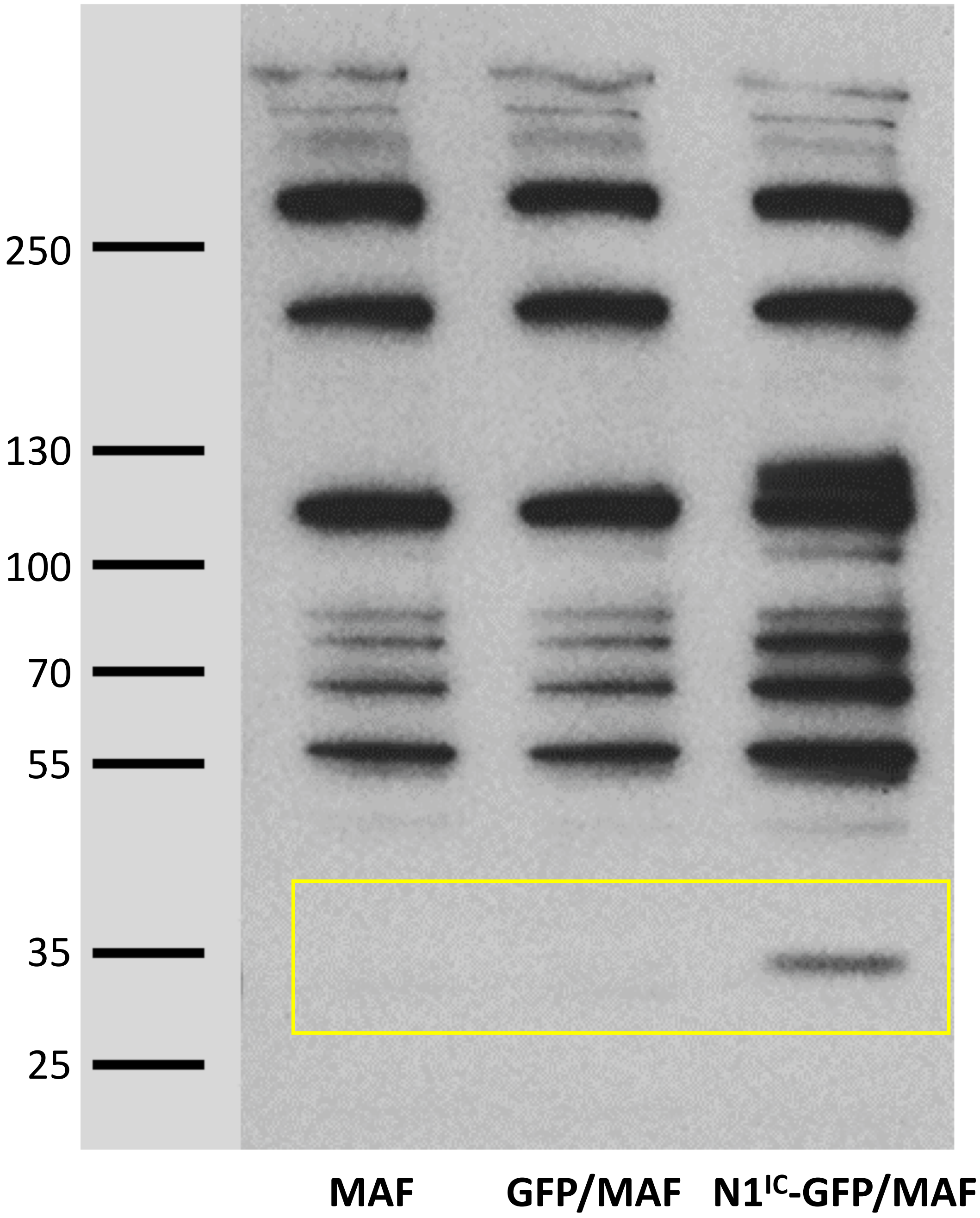

Fig. 3A β-actin 42kDa

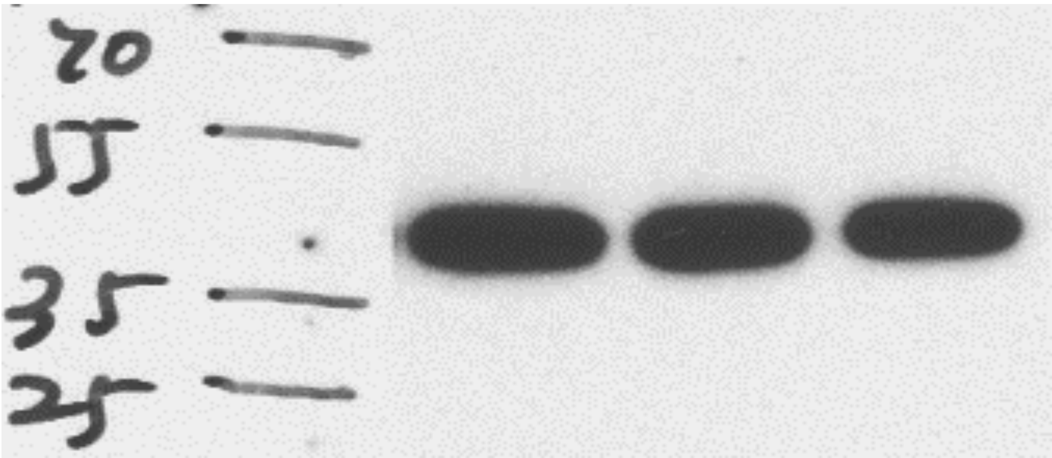

Cropped

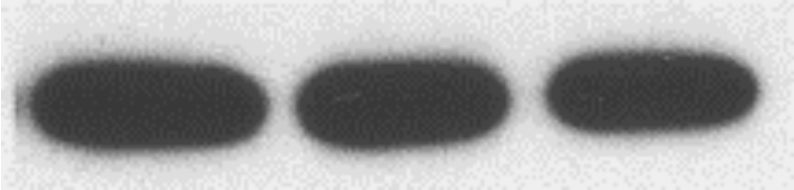

compressed
to fit space.

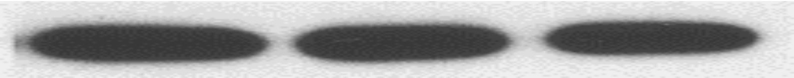

Fig. 4B WISP1: 40 kDa and 30.7/30.8kDa variants

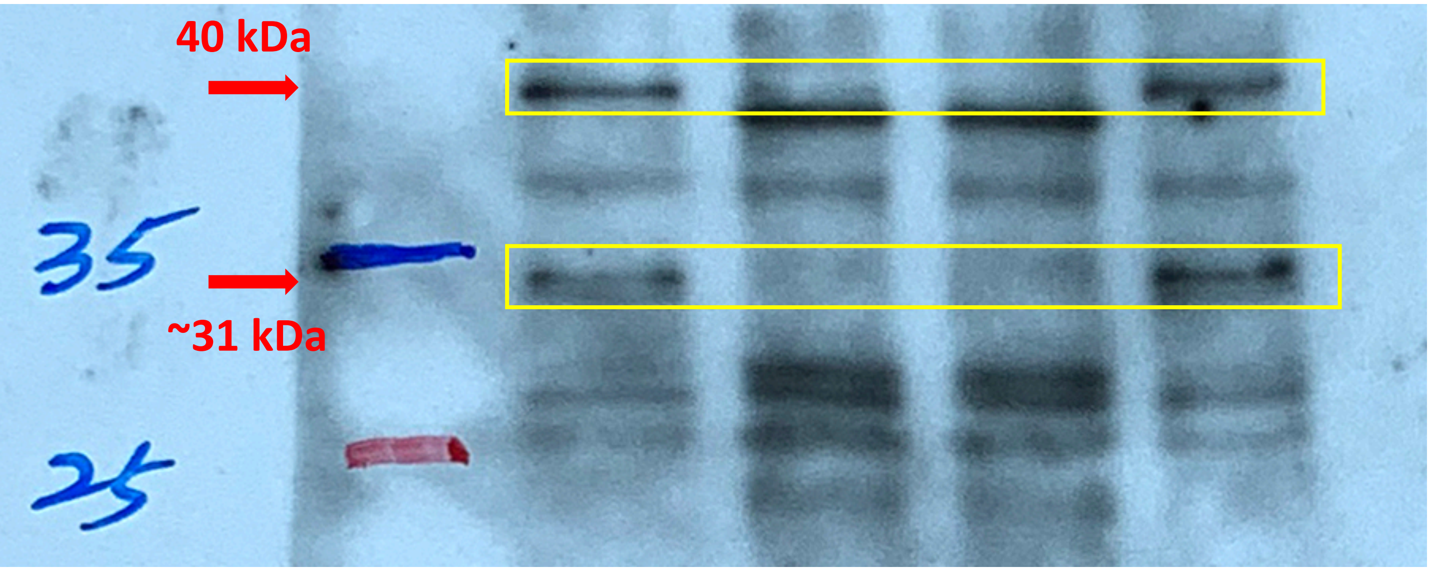

HDF **MAF** **GFP/MAF** **$N1^{IC}$-GFP/MAF**

We squeezed the original plot to fit into the very limited space in Figure 4.

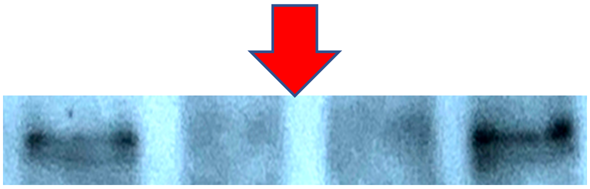

Fig. 4B β-actin 42kDa

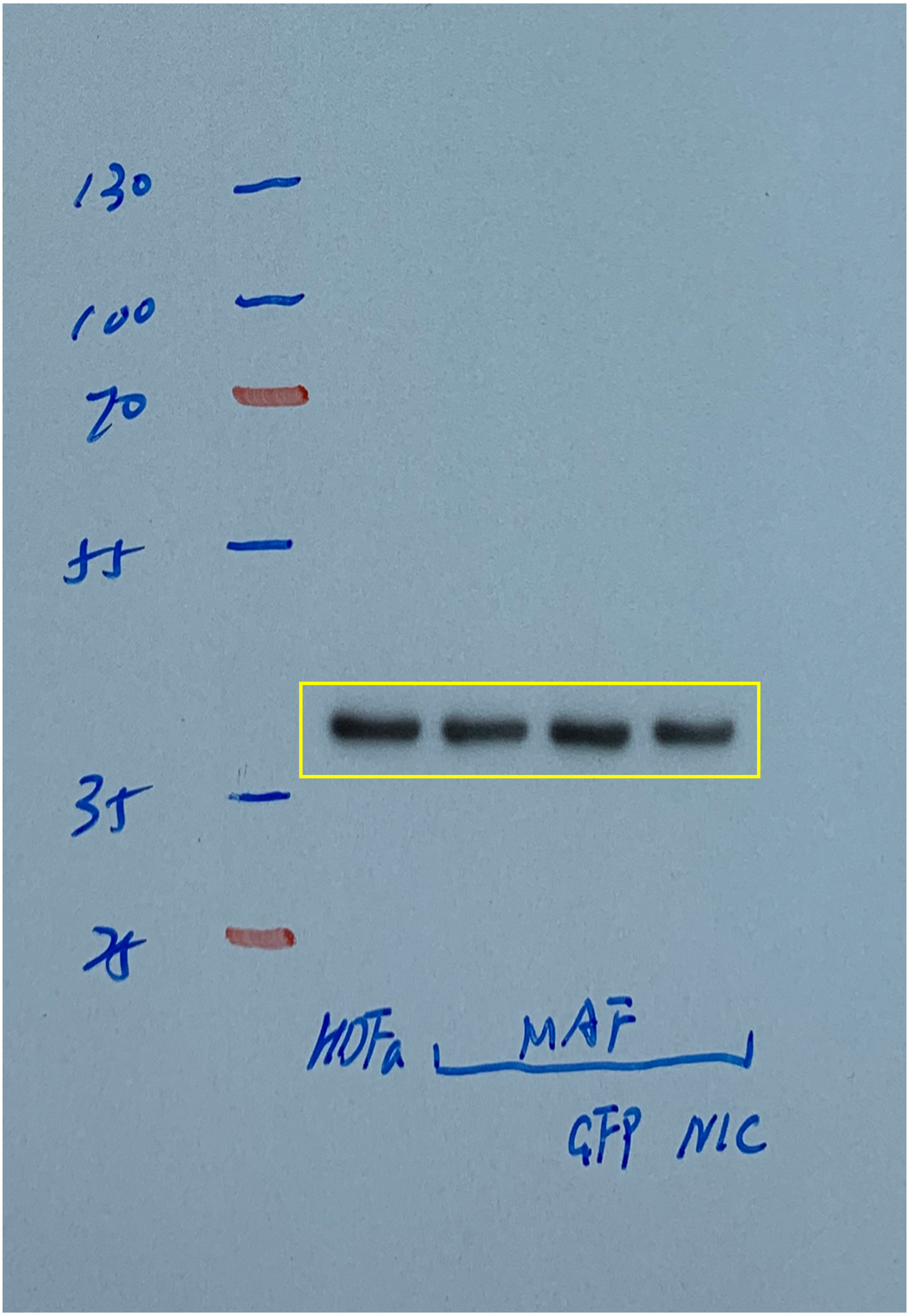

Suppl Fig. 6A MAML1 52kDa

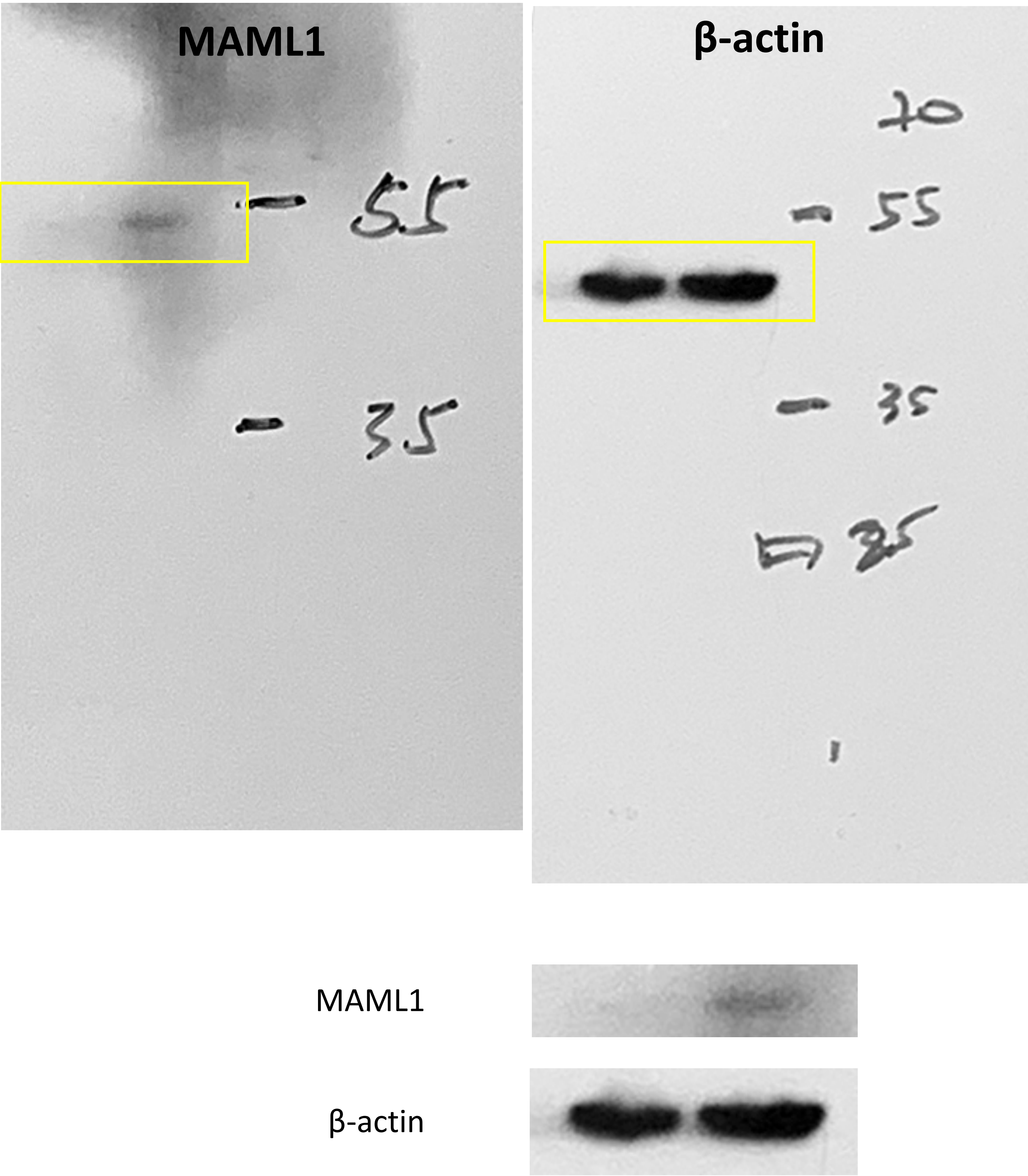

Supplement: S1 File — (PDF) [file pone.0248260.s008.pdf]
